# Proteome characterization of cassava (*Manihot esculenta *Crantz) somatic embryos, plantlets and tuberous roots

**DOI:** 10.1186/1477-5956-8-10

**Published:** 2010-02-27

**Authors:** Kaimian Li, Wenli Zhu, Kang Zeng, Zhenwen Zhang, Jianqiu Ye, Wenjun Ou, Samrina Rehman, Bruria Heuer, Songbi Chen

**Affiliations:** 1 Tropical Crops Genetic Resources Institute, Chinese Academy of Tropical Agricultural Sciences, Hainan Province, China; 2 Manchester Interdisciplinary Biocentre, University of Manchester, Manchester, UK; 3 Institute of Soil, Water and Environmental Sciences, ARO, Volcani Center, Bet-Dagan, Israel

## Abstract

**Background:**

Proteomics is increasingly becoming an important tool for the study of many different aspects of plant functions, such as investigating the molecular processes underlying in plant physiology, development, differentiation and their interaction with the environments. To investigate the cassava (*Manihot esculenta *Crantz) proteome, we extracted proteins from somatic embryos, plantlets and tuberous roots of cultivar SC8 and separated them by sodium dodecyl sulfate polyacrylamide gel electrophoresis (SDS-PAGE).

**Results:**

Analysis by liquid chromatography-electrospray ionisation-tandem mass spectrometry (LC-ESI-MS/MS) yielded a total of 383 proteins including isoforms, classified into 14 functional groups. The majority of these were carbohydrate and energy metabolism associated proteins (27.2%), followed by those involved in protein biosynthesis (14.4%). Subsequent analysis has revealed that 54, 59, 74 and 102 identified proteins are unique to the somatic embryos, shoots, adventitious roots and tuberous roots, respectively. Some of these proteins may serve as signatures for the physiological and developmental stages of somatic embryos, shoots, adventitious roots and tuberous root. Western blotting results have shown high expression levels of Rubisco in shoots and its absence in the somatic embryos. In addition, high-level expression of α-tubulin was found in tuberous roots, and a low-level one in somatic embryos. This extensive study effectively provides a huge data set of dynamic protein-related information to better understand the molecular basis underlying cassava growth, development, and physiological functions.

**Conclusion:**

This work paves the way towards a comprehensive, system-wide analysis of the cassava. Integration with transcriptomics, metabolomics and other large scale "-omics" data with systems biology approaches can open new avenues towards engineering cassava to enhance yields, improve nutritional value and overcome the problem of post-harvest physiological deterioration.

## Background

Cassava (*Manihot esculenta *Crantz) is a perennial woody shrub of the Euphorbiaceae native to South America that is extensively cultivated as an annual crop in tropical and subtropical regions for its edible starchy tuberous root, a major source of carbohydrates. Currently, cassava is the largest source of carbohydrates for human food in the world, and the world's sixth food crop for more than 700 million people in the tropics and sub-tropics. It has a high growth rate under optimal conditions and the tuberous roots as well as the leaves are used as human food, animal feed and industrial products [1-4]. Cassava roots combine high energy and high levels of some vitamins, minerals and dietary fiber, and contain no trypsin inhibitor [5], but create a problem due to presence of cyanide which is removed by post-harvest treatments and cooking. The edible green leaves of cassava are a good source of protein, vitamins and minerals and are often used to augment the rural diet [6]. Despite its importance, the research to improve cassava has lagged behind that of other crops such as rice, wheat, maize, and potatoes. Therefore, only relatively minor increases in cassava's productivity were obtained. Cassava breeding faces several limitations such as the crop's heterozygous genetic makeup which makes it time consuming to breed efficiently [7] and the parental lines used to generate new segregating progenies makes it difficult to identify the parents with good breeding values. Few studies have been published, therefore, the cassava breeder has to work without the advantages of a clear understanding of the way the traits to be improved are inherited [8].

As for all crop species, transgenic systems in cassava are reliant on the development of tissue culture systems capable of generating totipotent cells and tissues [9]. This cultivation method enables reproducibility of the plant material providing a plant biomass that can be maintained aseptically, facilitating the performance of perfectly controlled experiments dealing with inoculation, propagation and pathogenicity of several plant pathogens [10]. Proliferating embryogenic somatic embryos clumps are known to regenerate easily and to have the potential for genetic transformation [11]. A large reproducible biomass can open additional avenues for basic and applied research.

The integration and expression of transgenes in cassava is rather limited, but it is generating important new knowledge [12]. In addition, technologies for plant regeneration and transformation are opening up new possibilities to generate improved cassava genotypes by integrating desired traits into farmer-preferred cultivars [13]. To date, transgenic biological technology has been integrated into cassava to reduce cyanogenic content, improve insect, virus, and herbicide resistance, manipulate starch content and elevate protein content for nutritional enhancement [14]. Furthermore, a range of selectable and visual marker genes have been tested and developed for use in cassava transformation systems, including GUS, luciferase and GFP visual marker systems, employed as tools for developing transgenic systems in cassava and investigating transgene expression pattern [14].

Successful exploitation of genomics tools and strategies in plant breeding programmes requires extensive and precise phenotyping of agronomic traits for breeding materials, mapping populations, natural populations and also for gene bank materials. Genomics research has successfully unravelled various metabolic pathways and provided molecular markers for agronomic traits. However, knowledge of inheritance patterns associated with traits of agronomic relevance in cassava is limited. The mechanisms of epigenetic phenomena are only beginning to be understood and their potential role in crop improvement is unknown [15]. In this study we extracted proteins from cassava cultivar SC8 somatic embryos, plantlets (adventitious roots and shoots) and tuberous roots. SDS-PAGE was performed for protein separation analysis and the nano-LC-ESI-MS/MS was used for protein identification. All proteins were clustered into cohesive groups based on their biochemical functions. The basis of our findings provides an insight into a high number of proteins identified and their relation with the wide range of physiological functions. It would lead to understand better the mechanism associated with the systematic breeding phenotype.

## Results & Discussion

**Figure 1:**
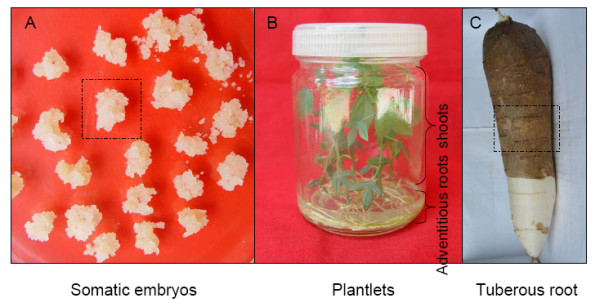
**Different developmental stages of cassava cultivar SC8**. A, The pure somatic embryos (SE). The framed region is a single SE. B, Plantlets. Protein extracts are from adventitious roots and shoots of plantlets, respectively; and C, Tuberous root. The framed region presents the place of protein extracts.

Previous studies on inducing primary and secondary somatic embryos and establishing a plant regeneration system for cassava have already been discussed [15-17]. Fig. 1 shows developmental stages of cassava cultivar SC8: somatic embryos separated from medium and prepared for protein extraction (Fig. 1A), adventitious roots and shoots from one month plantlets in tissue-culture (Fig. 1B) and the midst of the tuberous root (Fig. 1C).

Protein content in cassava tuberous roots is low, between 1 to 2%. By enhancing protein levels in these roots, cassava might become more nutritious and also increase its value as animal feed [7]. The breakthrough of high-throughput tools (For example, DNA finger printing, DNA sequencing, DNA and protein biochips, two-dimensional gel combining with mass spectrometry and so on) [18,19] in advanced molecular and cell biology offers new approaches that may vastly improve our understanding and facilitate the challenges we face to cassava breeding limitations. Combining these new technologies and complementing with conventional plant breeding may yield a much more productive and nutritious cassava genotype, which will be profitable to grow.

**Figure 2:**
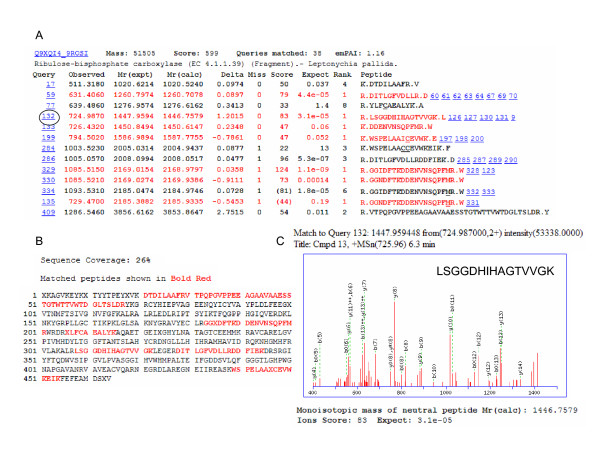
**Results of Rubisco in cassava cultivar SC8 tuberous roots as a representation using LC-ESI-MS/MS**. A and B, Output of the database searching by the MSCOT program using MS/MS data in the identification of Rubisco. The matched peptides were shown in bold red and sequence coverage was 26%. C. MS/MS spectrum of ions with *m/z *values of 725.96 in panel A covered with circle.

**Figure 3:**
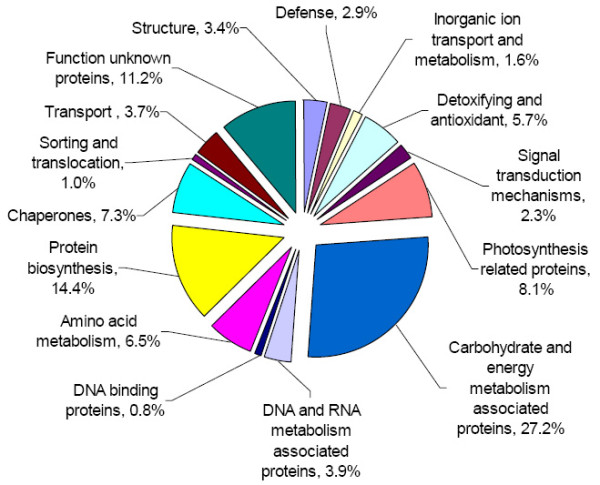
**The functional classification and distribution of all 383 identified proteins from cassava cultivar SC8**. Unknown proteins include those whose functions have not been described.

In order to further analyze expressed protein patterns in cassava, we separated proteins from somatic embryos, plantlets and tuberous roots with 4-15% gradient gels, and identified them by LC-ESI-MS/MS. We endeavoured to capture the entire proteomic profiles, and have included all findings in the additional files 1, 2, 3, and 4, Tables, S1-4, although the possibility could not be excluded that some proteins expressed in minute quantities were not retained or not identified in the present analysis. The proteins identified by LC-ESI-MS/MS in this study (albeit a small number) were highly abundant. We identified 112 proteins in somatic embryos, 110 proteins in shoots, 147 proteins in adventitious roots and 155 proteins in tuberous roots, i.e. a total of 524 proteins (including their isoforms). So far the cassava genome testing work is not done yet; thus it is impossible to determine the percentage of our data from the total cassava proteome. During identification, many repeat proteins were detected and deleted. As a result, we ended with a final number of 383 proteins (Additional files, 1, 2, 3, and 4, Tables, S1-4). The 383 identified proteins were annotated according to gene ontology, including their predicted functions. One example of MS/MS spectra is presented in shoots as a representation using LC-ESI-MS/MS (Fig. 2). These peptides covered 26% of the protein sequence (Fig. 2B). Panel C represents an MS/MS spectrum of ions with *m/z *values of 725.96, which led to the identification of a unique protein, Rubisco (Fig. 2A). Another representation of MS/MS spectra from adventitious roots is shown in additional file 5, Figure S1. Only one peptide covered 7% of the protein sequence, which led to the identification of a unique protein, mitochrondrial voltage-dependent anion-selective channel. Details of the additional proteins identified using LC-ESI-MS/MS are available in additional files, 1, 2, 3, and 4, Tables, S1-4. The 383 proteins including their isoforms were classified into 14 groups based on their biochemical functions [3,20-22], and their distribution is shown in Fig. 3. The majority of these were carbohydrate and energy metabolism associated proteins (27.2%), followed by protein biosynthesis (14.4%), unknown function proteins (11.2%), photosynthesis related proteins (8.1%) and chaperones (7.3%). Only 10 proteins were found in all the tissues tested (2.6%) (Table 1 and 2).

**Table 1:** Protein classification in somatic embryos, plantlets and tuberous roots.

Function	Total proteins	Exist in all tissues	Exist in plantlet roots and tuberous roots	Unique to somatic embryos	Unique to shoots	Unique to adventitious roots	Unique to tuberous roots
Structure	13	2	3	1	2	2	3
Defense	11			1	1	5	2
Inorganic ion transport and metabolism	6		2	1		1	
Detoxifying and antioxidant	22	1	4		4	6	6
Signal transduction mechanisms	9		1	1	1	3	1
Photosynthesis related proteins	31				21	3	4
Carbohydrate and energy metabolism associated proteins	104	4	13	12	16	20	33
DNA and RNA metabolism associated proteins	15		1	6		4	3
DNA binding proteins	3			1			
Amino acid metabolism	25		2	3	2	8	6
Protein biosynthesis	55	1	5	13	3	10	7
Chaperones	28		1	5	3	6	6
Sorting and translocation	4		1	1		1	1
Transport	14			7	3	1	3
Function unknown proteins	43	2	4	2	3	4	27
***Total proteins***	***383***	***10***	***37***	***54***	***59***	***74***	***102***

**Table 2:** Proteins existed in somatic embryos, plantlets and tuberous roots.

Protein name	Accession no^*a*^	Theoretical molecular mass (KDa)/pI^*b*^
***Structure *(2)**		
Actin - *Gossypium hirsutum *(Upland cotton)	Q7XZI7_GOSHI	41.701/5.31
Alpha-tubulin 4 (Fragment) - *Gossypium hirsutum *(Upland cotton)	Q8H6L8_GOSHI	34.013/5.36
***Detoxifying and antioxidant *(1)**		
Ascorbate peroxidase APX3- *Manihot esculenta *(Cassava) (Manioc)	Q52QX1_MANES	27.652/5.31
***Carbohydrate and energy metabolism associated proteins (4)***		
Enolase - *Gossypium barbadense *(Egyptian cotton)	Q6WB92_GOSBA	47.702/6.16
Fructose-bisphosphate aldolase, cytosolic - common ice plant	T12416	38.134/6.49
Malate dehydrogenase, cytosolic - common ice plant	T12433	35.475/6.00
H+-transporting two-sector ATPase beta chain, mitochondrial - Para rubber tree	S20504	60.221/5.95
***Protein biosynth (1)***		
Elongation factor 1-alpha - *Zea mays *(Maize)	O50018_MAIZE	49.259/9.19
***Function unknown proteins (2)***		
AF255338 NID - *Glycine max*	AAF70292	25.964/4.70
Arabidopsis thaliana genomic DNA, chromosome 5, P1 clone:MEE6 - *Arabidopsis thaliana *(Mouse-ear cress)	Q9FLL1_ARATH	66.684/5.55
**The total protein number (10)**		

a, MSDB accession number. b, Theoretical molecular mass (kDa) and pI from the MSDB database.

The largest functional group in this study was associated with carbohydrate and energy metabolism proteins and composed of 104 proteins (Table 1). Thirty three unique proteins from tuberous roots compared with 20 from adventitious roots, 12 from somatic embryos, and 16 from plantlet shoots indicated that active metabolism takes place in tuberous roots (Additional file 6, Table S5). ATP is an energy source produced by a number of distinct cellular processes; the three main pathways used to generate energy in eukaryotic organisms are glycolysis in the cytosol and the citric acid cycle (the Krebs cycle)/the oxidative phosphorylation in the mitochondria, both components of cellular respiration; and photophosphorylation in the tylakoid membranes of the chloroplasts. The activity of the citric acid cycle enzymes is controlled by the energy status of the cell. Thus, the enzymes related to the glycolysis and citric acid cycle are correlated with the cellular ATP utilization in higher plants [22]. Sheffield and his colleagues used proteomics as a tool to analyze the proteome involved in cassava fibrous and tuberous root tissues and obtained 232 differential proteins [3]. Their quantitative and qualitative analysis data could be helpful to characterize the differentially expressed proteins. In this study, we extended the proteome research to somatic embryos, adventitious roots, shoots and tuberous roots and identified all tissue proteins. We firstly reported the characterization of cassava somatic embryo proteome. In addition, this extensive study provides a huge data set of proteins that are present in cassava, may help to improve the understanding of the metabolic processes within cassava breeding and thus pave the way to further studies on the resistance to extreme environments. We report the existence of a large group of proteins related to carbohydrate metabolism and energy production: 1,4-alpha-glucan branching enzyme precursor, starch branching enzyme I, starch phosphorylase isoform L precursor, starch phosphorylase L, starch phosphorylase precursor, ADP-glucose pyrophosphorylase associated with starch metabolism [12,23,24], all of which are unique to tuberous roots of cassava (Additional file 6, Table S5), indicating that tuberous roots are not only for storage, but also metabolically active.

The cassava root system is distinguished by different adventitious root types: fibrous roots and tuberous roots. Fibrous roots absorb water and mineral salts. In addition, they provide a support function. The tuberous roots accumulate starch as a reserve compound [25], these are vegetative structures, and have none of the reproductive properties associated with other storage organs such as the potato tubers. Cassava tuberous roots develop from fibrous roots through massive cell division and differentiation of parenchyma cells of the secondary xylem [3,26]; however, not all fibrous roots are designated for tuberous root formation. Little is known about the mechanism involved in the transition from fibrous roots to tuberous roots. Sheffield and his colleagues (2006) compared the proteome of cassava fibrous and tuberous roots and tried to unravel the molecular differences between the two types of roots; 2 unique proteins to fibrous roots and 14 proteins to tuberous roots were found in 232 differentially identified proteins [3]. In this study, we concentrated on the protein pattern of both roots and analyzed all protein composition. We have identified 147 proteins present in cassava adventitious roots (Additional file 3, Table S3), and 155 proteins in tuberous roots (Additional file 4, Table S4). Further analysis showed that a total of 37 proteins were present in both adventitious and tuberous roots (Table 3), 74 unique proteins to adventitious roots and 102 unique proteins to tuberous roots (see Table 1 and Additional file 6, Table S5), indicating that the two types of roots have both overlapping and different metabolic activities.

**Table 3:** Proteins existed in adventitious roots and tuberous roots.

Protein name	Accession no^*a*^	Theoretical molecular mass (KDa)/pI^*b*^
***Structure (3)***		
Actin - *Gossypium hirsutum *(Upland cotton)	Q7XZI7_GOSHI	41.701/5.31
Alpha-tubulin 4 (Fragment) - *Gossypium hirsutum *(Upland cotton)	Q8H6L8_GOSHI	34.013/5.36
Tubulin beta chain - *Chlamydomonas reinhardtii*	UBKM	49.587/4.82
***Inorganic ion transport and metabolism (2)***		
Mitochrondrial voltage-dependent anion-selective channel - *Phaseolus coccineus *(Scarlet runner bean)	Q4PKP6_PHACN	29.710/8.56
Outer plastidial membrane protein porin (Voltage-dependent anion- selective channel protein) - *Pisum sativum *(Garden pea)	VDAC_PEA	29.448/9.11
***Detoxifying and antioxidant (4)***		
Ascorbate peroxidase APX3 - *Manihot esculenta *(Cassava) (Manioc)	Q52QX1_MANES	27.652/5.31
Catalase CAT1 - *Manihot esculenta *(Cassava) (Manioc)	Q9SW99_MANES	57.137/6.87
Malic oxidoreductase - *Medicago truncatula *(Barrel medic)	Q1SRX6_MEDTR	65.296/5.95
Monodehydroascorbate reductase - *Mesembryanthemum crystallinum *(Common ice plant)	Q93YG1_MESCR	51.716/6.38
***Signal transduction mechanisms (1)***		
Cytokinin binding protein CBP57 - *Nicotiana sylvestris *(Wood tobacco)	Q42939_NICSY	49.227/6.10
***Carbohydrate and energy metabolism associated proteins (13)***		
Aldolase (Fragment) - *Triticum aestivum *(Wheat)	Q7X9K7_WHEAT	23.183/8.90
Enolase - *Gossypium barbadense *(Egyptian cotton)	Q6WB92_GOSBA	47.702/6.16
Fructose-bisphosphate aldolase, cytosolic - common ice plant	T12416	38.134/6.49
Glyceraldehyde-3-phosphate-dehydrogenase - *Lupinus albus *(White lupin)	Q53I52_LUPAL	32.166/6.80
Malate dehydrogenase, cytosolic - common ice plant	T12433	35.475/6.00
Phosphoglycerate kinase, putative - *Arabidopsis thaliana *(Mouse-ear cress)	Q8LFV7_ARATH	42.121/5.49
Sucrose synthase (Fragment) - *Manihot esculenta *(Cassava) (Manioc)	Q5PYQ4_MANES	31.489/5.44
Transaldolase-like- *Solanum tuberosum *(Potato)	Q2XTB7_SOLTU	47.883/5.95
Transketolase precursor, chloroplast - spinach	T09015	80.231/6.20
UDP-glucose pyrophosphorylase - *Populus tremula *× *Populus tremuloides*	Q5YLM4_9ROSI	51.778/5.68
AAA family ATPase, CDC48 subfamily - *Oryza sativa *(japonica cultivar-group)	Q7XE16_ORYSA	90.857/5.07
H+-transporting two-sector ATPase beta chain, mitochondrial - Para rubber tree	S20504	60.221/5.95
NADH dehydrogenase subunit F (Fragment) - *Typha angustifolia *(Narrow leaf cattail)	O47212_TYPAN	77.016/9.13
***DNA and RNA metabolism associated proteins (1)***		
Maturase-like protein - *Adesmia volckmannii*	Q9TKT4_9FABA	61.146/8.98
***Amino acid metabolism (2)***		
Methionine synthase (Fragment) - *Coffea arabica *(Coffee)	Q9M619_COFAR	24.430/5.69
S-adenosyl-L-methionine synthetase - *Beta vulgaris *(Sugar beet)	Q4H1G4_BETVU	43.189/5.57
***Protein biosynthesis (5)***		
Elongation factor 1, gamma chain - *Medicago truncatula *(Barrel medic)	Q1SL16_MEDTR	47.694/6.43
Elongation factor 1-alpha - *Zea mays *(Maize)	O50018_MAIZE	49.259/9.19
Elongation factor Tu - *Medicago truncatula *(Barrel medic)	Q1S824_MEDTR	94.123/5.91
Ribosomal protein S3 - Norway spruce chloroplast	T11807	25.380/9.62
Translation elongation factor eEF-2 - beet	T14579	93.738/5.93
***Chaperones (1)***		
Chaperonin groEL - castor bean (fragment)	HHCSBA	52.347/4.77
***Sorting and translocation (1)***		
Pollen coat oleosin-glycine rich protein - *Cardaminopsis arenosa *(Arabidopsis arenosa)	Q6V5C0_CARAS	15.788/10.54
***Function unknown proteins (4)***		
AF255338 NID - *Glycine max*	AAF70292	25.964/4.70
Arabidopsis thaliana genomic DNA, chromosome 5, P1 clone:MEE6 - *Arabidopsis thaliana *(Mouse-ear cress)	Q9FLL1_ARATH	66.648/5.55
Hypothetical protein - *Citrus paradisi *(Grapefruit)	O04428_CITPA	32.623/5.46
OSJNBa0067K08.13 protein - *Oryza sativa *(japonica cultivar-group)	Q7XUK3_ORYSA	37.537/6.28
***The total protein number *(37)**		

a, MSDB accession number. b, Theoretical molecular mass (kDa) and pI from the MSDB database.

**Figure 4:**
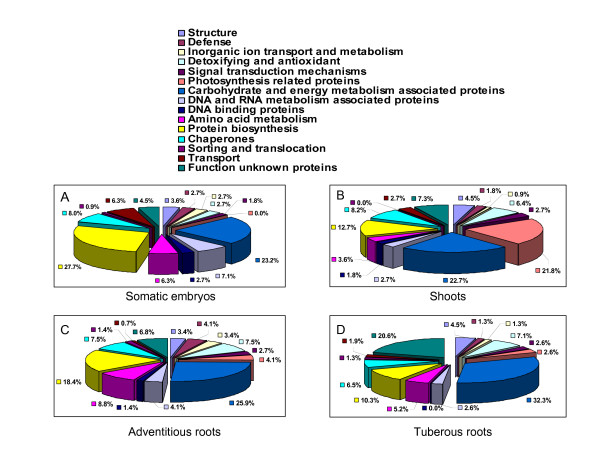
**The functional classification and distribution of identified proteins from somatic embryos, adventitious roots, shoots and tuberous roots**.

**Figure 5:**
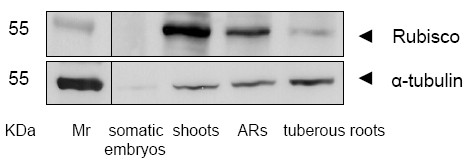
**Western blotting of Rubisco and α-tubulan**. Rubisco and α-tubulan in the somatic embryos, plantlets and tuberous roots of cassava cultivar SC8 were detected by Western blotting using antiRubisco-polyclonal antibody from Agrisera (AS07218) and anti-α-tubulin-monoclonal antibody from Sigma-Aldrich (T9026). ARs, adventitious roots.

The proteins involved in photosynthesis were among the largest group identified in shoots (21.8%) (Fig. 4B), but only 4.1% of these were presented in adventitious roots (Fig. 4C) and 2.6% were presented in tuberous roots (Fig. 4D). No differentiation in proteins involved in photosynthesis were observed in somatic embryos (Fig. 4A). Photosynthetic enzymes are abundantly expressed in green tissues. Among them, the most abundant and important one is Rubisco, which represents about 50% of the total protein content in leaves [21,22]. The results from western blots indicated high Rubisco levels in shoots and its absence in somatic embryos, suggesting Rubisco may be among the controlled keys of the photosynthetic pathways (Fig. 5 and Additional file 7, Figure S2). We assume that chlorophyll a/b-binding protein, oxygen-evolving enhancer protein 1 (OEE1), are involved in photosynthesis and unique to shoots and adventitious roots (Additional file 6, Table S5), and that these may be synthesized in the shoots and then transported to the roots. Similar results were found in the proteome profile of tomato seedlings [21].

Proliferation embryogenic callus clumps are known to regenerate easily and to have the potential for genetic transformation. Cassava plants recovered from somatic embryos have been reported [17,27]. In this study, we have firstly characterized the cassava somatic embryo proteome and analyzed the protein pattern, as shown in Fig. 4A and Table 1. Interestingly, the largest functional group (27.7%) in somatic embryos was of proteins involved in protein biosynthesis. Further analysis of this group in shoots (12.7%) (Fig. 4B), adventitious roots (18.4%) (Fig. 4C) and tuberous roots (10.3%) (Fig. 4D), suggest that somatic embryogenesis could be possible closely associated with protein biosynthesis metabolism.

Microtubules have a unique role in plant morphogenesis, and may influence both, morphology of individual cells and the entire plant. The influence of plant microtubules on morphology is attributable to their involvement in establishing cellular division planes, in determining the axes of cellular elongation, and in regulating the deposition of cellulose microfibrils in the cell wall [28]. The building block of a microtubule is the tubulin subunit, a heterodimer of α- and β-tubulin. In this study the results from western blot suggest that the expressed level of α-tubulin involved in cell structure, is different in somatic embryos, shoots, adventitious roots and tuberous roots (Fig. 5 and Additional file 7, Figure S2). The highly expressed α-tubulin in tuberous roots, and the low-level expression observed in somatic embryos indicate that fiber development in tuberous roots is high and by contrast low in somatic embryos.

In this study we investigated the proteomic patterns in somatic embryos, plantlets and tuberous roots and showed 54, 59, 74 and 102 identified proteins are unique to somatic embryos, shoots, adventitious roots and tuberous roots, respectively (Table 1). Details of these proteins are available in additional file 6, Table S5. Further analysis of these protein patterns were performed by investigating into the protein fractions within the somatic embryos, plantlets and tuberous roots with LabChip GXII (see Appendix 1). The LabChip analysis of all separated fractions revealed different patterns of proteins presented in these tissues. This information is given in additional file 8, Figure S3 A&B. The left lane represented the molecular mass markers (Caliper LifeScience). The somatic embryo fraction covered relatively small masses between 10 to 70KDa. Fractions in adventitious roots, shoots and tuberous roots contained highly expressed polypeptides; e.g. 11.10 and 56.85 KDa in adventitious roots, 57.12, 27.19, 15.23, and 10.89KDa in shoots, and 107.54, 35.93-42.63, 26.65, 23.80, 20.80, 15.67KDa in tuberous roots.

In our study the proteome profile of cassava and its characterization provided a foundation for understanding the molecular basis underlying in cassava growth, development, and physiological functions, and thus will open new avenues towards engineering cassava for enhanced yields and improved nutritional value. These data combined with previous findings may provide the means for integration and structured interrogation of datasets that will facilitate the cross-fertilization of disciplines [15]. However, a holistic system-wide understanding of the cassava may be the way forward to address the challenges in cassava production. Such a comprehensive understanding of the cassava cell system may be achieved using systems biology approaches. This paradigm would attempt to harness the power of mathematics, engineering, and computer science to analyze and integrate data from all the 'omics' studies and ultimately lead to creating a complete understanding of how all components of cassava work together to maintain and procreate life. By quantitatively profiling one at a time, the effect of thousands and millions of genetic and environmental perturbations on the cassava cell, systems biology approaches can attempt to recreate and measure the effect of the many different states that cassava has/would be exposed to under normal and stress conditions. A key aspect of this work would involve the development of innovative new approaches to quantify changes in the transcriptome, proteome, and metabolome of cassava.

## Conclusions

In this study we show the power of the proteomic approach in cassava biology studies and give a new insight into investigating the proteomic patterns in the somatic embryos, plantlets and tuberous roots. In addition, this investigation revealed the presence of approximately 383 proteins. Subsequent analysis has shown 54, 59, 74 and 102 identified proteins are unique proteins to the somatic embryos, shoots, adventitious roots and tuberous roots, respectively. Some of these proteins may serve as signatures for the physiological and developmental stages of somatic embryos, plantlets and tuberous root organs as well as potential targets for further biochemical and physiological characterizations. This study will not only facilitate an insight into the molecular processes underlying cassava physiology and differentiation, but will also be valuable for engineering cassava for better disease resistance, higher yields and improved nutritional quality.

## Methods

### Cassava Tissue Culture

Cassava cultivar SC8 was obtained from the *in vitro *Germplasm Collection Pool, Tropical Crops Genetic Resource Institute (TCGRI), Chinese Academy of Tropical Agricultural Sciences (CATAS). The plantlets were obtained from the shoot, which was used as monthly subculture *in vitro *as previously described [29]. Shoot apical meristem and immature leaf lobe (0.1-1.0 mm long) were cultured on CIM medium (1× MS salts with vitamins, 12 mg l^-1 ^picloram, 2 M CuSO4, 2% sucrose and 0.3% Gelrite; pH of the medium was adjusted to 5.8 before autoclaving) for the induction of primary somatic embryos [30]. Leaf lobes and meristem cultures were incubated in the dark for one week, and then, transferred to the light for a further three weeks until the production of primary somatic embryos was observed. Primary somatic embryos could be induced to produce secondary somatic embryos by further subculturing on CIM medium every second week. By keeping constant subculture of somatic embryos, a cyclic embryogenesis system was established. After three cycles, somatic embryo clusters of SC8 were moved to GD medium (1× GD salts with vitamins, 12 mg l^-1 ^picloram, 2% sucrose, 0.3% Gelrite; pH of the medium was adjusted to 5.8 before autoclaving) and cultured at 26°C in the dark, then the three-month-old FEC suspension, (FEC, a small globular embryo like structure with a light yellow colour), was separated and placed on GD medium at 26°C under a 16-hour photoperiod. After 2 weeks, a pure FEC was selected and transferred to a fresh GD medium for subculture.

### Protein Extraction and Separation

Protein extracts from cassava somatic embryos, plantlets and tuberous roots were performed according to Chen et al. 2006 [31]. In this study, three independent analyses were performed. In each independent analysis we grew somatic embryos with 3 Petri dishes, plantlets with 6 bottles which contained 5 plantlets, and three different tuberous roots taken from three different cassava plants. From each independent protein extraction, we collected 1.0 g plant material for the proteomic analysis. Proteins prepared for SDS-PAGE were dissolved in SDS sample buffer (50 mM Tris · Cl, pH 6.8, 100 mM dithiothreitol, 2% SDS, 10% glycerol) and boiled at 100°C for 10 minutes. The supernatant was collected after centrifugation at 10,000 g for 5 minutes. Protein contents were quantified according to the principles and methods of Bio-Rad protein assay (Catalog 500-0006, Bio-Rad laboratories, Hercules, CA, USA) using BSA as a standard. Proteins were separated on a 4-15% gradient gel (Bio-RAD), and stained with Colloidal Coomassie Blue G-250.

### Tryptic In-gel Digestion

Briefly, the SDS-PAGE gels were incubated with 1.5 mM DTT for 40 minutes, then transferred to 3.0 mM iodoacetamide for a further 40 minutes and washed with MillQ water for 5 min. The gel lanes were excised and cut horizontally into 12 sections of similar size. Excised sections were cut into ~1-mm^3^, then, washed twice in MillQ water for 15 min. The washed gel pieces were subjected to two cycles of dehydration with 50% acetonitrile followed by rehydration with 50 mM ammonium bicarbonate solution for 15 min per cycle and digested overnight at 37°C in 20 μL of sequencing grade trypsin (Sigma-Aldrich) according to the manufacturer's instructions (1 μg in 100 μL of 50 mM ammonium biocarbonate). The supernatants were transferred to a fresh tube and stored at room temperature until required. 30 μL MilliQ water was added to the gel pieces at room temperature for 1 hour. Following this, the two supernatants were pooled together.

### Protein Identification Using LC-ESI MS and Database Search

Mass spectrometric analyses were conducted by nanoflow-LC-ESI-MS/MS (Bruker Esquire 3000plus Ion Trap; Bruker Daltonics). Gel lanes excised from SDS-PAGE gels and cut into 1-mm^3 ^were digested with trypsin and analyzed by LC-MS/MS. The LC-MS/MS analysis was carried out as described [32]. Peptides were separated by chromatography on a 75 μm × 15 cm PepMap nanocolumn (LC Packings) at a flow rate of 250 nL/min using a linear gradient of acetonitrile (5-95% in 60 min) in 0.1% formic acid. The column effluent was sprayed directly into the ion trap which was set to scan the m/z rang from 400 to 1500 in positive ion mode, capturing MS and MS2 data automatically. Instrument operation, data acquisition, and analyses were performed using HyStarTM V2.3 and DataAnalysis V3.1 software.

Data captured by either LC-ESI-MS/MS were matched using the MASCOT version 2.2.03 (Matrix Science, UK http://www.matrixscience.com) against MSDB (MSDB database update Sep 08 2006, 3239079 sequences; Taxonomy: Viridiplantae (Green Plants), 247835 sequences). Carbamidomethyl (Cys) and oxidation (Met) were considered as variable modifications and a single missed cleavage was permitted. For LC-MS/MS data, peptide mass tolerance was set as 3.0 Da and MS/MS ion mass tolerance was set at 1.5 Da. Peptide charge states (+1, +2, +3) were taken into account. Routine protein identification required sequence-confirmed data for a minimum of one peptides with recognition as the top ranking match in the Mascot Standard scoring system [32].

### Western Blot

Samples from cassava somatic embryos, plantlets and tuberous roots prepared for SDS-PAGE were homogenized and dissolved in SDS sample buffer (50 mM Tris · Cl pH 6.8, 100 mM dithiothreitol, 2% SDS, 10% glycerol). Ice-cold acetone was added 4 times to precipitate in ice for 1 h. This was then centrifuged at 5,000 g for 5 min. The pellet was air dried for 30-60 min then dissolved in 100 μ1 SDS sample buffer, and boiled at 100°C for 10 minutes, then centrifuged at 13,000 g for 5 min to remove debris. Fixed amounts of protein were separated by gel electrophoresis, blotted on to nitrocellulose (N8017-5EA, Sigma-Aldrich) and proteins detected by immuno-staining with the following antibodies: antiRubisco-polyclonal antibody from Agrisera (AS07218) and anti-α-tubulin-monoclonal antibody from Sigma-Aldrich (T9026). Western blots were developed using the principles and methods of NBT/BCIP from Roche (11 681 451 001).

## Abbreviations

ATP: adenosine-5'-triphosphate; LC-ESI-MS: liquid chromatography/electrospray ionization mass spectrometer; PAGE: ployacrylamide gel electrophresis; Rubisco: ribulose-1, 5-bisphosphate carboxylase.

## Competing interests

The authors declare that they have no competing interests.

## Authors' contributions

SC made the major contributions to this study in the conception, design, drafting manuscript, final revision and part of protein identification. KL contributed to part of design and protein identification. WZ worked at the somatic embryo tissue cultures, protein extraction and drafting part of manuscript and part of protein data analysis. ZK participated to part of design and protein data analysis. ZZ worked at plantlet cultures and protein data analysis. JY worked at LabChip protein separation. WO worked at part of protein data analysis. SR contributed to the methodology e.g. Tryptic In-gel Digestion and Protein Identification Using LC-ESI-MS/MS and Database Search and revised manuscript. BH was involved in the manuscript critical revision and given final approval of the version to be published. All authors read and approved the final manuscript.

## Appendix 1

### LabChip Protein Separation

Protein separation from somatic embryos, plantlets (adventitious roots and shoots) and tuberous roots was performed using the principles and methods of LabChip GXII (Caliper LifeScience). Based on a microfluidic version of SDS-PAGE, the LabChip GXII Electrophoresis System uses a single sipper microfluidic chip to aspirate protein samples directly from 96-well plates. The microfluidics chip technology automatically stains, destains, and electrophoretically separates the protein samples. System software, LabChipGX program (Version 1.1.119.0), automatically analyzes the data and provides the user with relative protein concentration, molecular size, and percent purity using ladder and marker calibration standards. Digital data results are immediately available for review or reporting in virtual gel, electropherogram graph, or table summary form. In this experiment 4 replicates were performed.

## Supplementary Material

Additional file 1 **Table S1.** Protein identification in cassava cultivars SC8 somatic embryos. a, MSDB accession number. b, Theoretical molecular mass (kDa) and pI from the MSDB database. c, Probability-based MOWSE (molecular weight search) scores. d, The number of unique peptides identified by MS/MS sequencing, and individual ions scores are all identity or extensive homology (p < 0.05). Click here for file

Additional file 2 **Table S2.** Protein identification in cassava cultivars SC8 shoots. a, MSDB accession number. b, Theoretical molecular mass (kDa) and pI from the MSDB database. c, Probability-based MOWSE (molecular weight search) scores. d, The number of unique peptides identified by MS/MS sequencing, and individual ions scores are all identity or extensive homology (p < 0.05). Click here for file

Additional file 3 **Table S3.** Protein identification in cassava cultivars SC8 adventitious roots. a, MSDB accession number. b, Theoretical molecular mass (kDa) and pI from the MSDB database. c, Probability-based MOWSE (molecular weight search) scores. d, The number of unique peptides identified by MS/MS sequencing, and individual ions scores are all identity or extensive homology (p < 0.05). Click here for file

Additional file 4 **Table S4.** Protein identification in cassava cultivars SC8 tuberous roots. a, MSDB accession number. b, Theoretical molecular mass (kDa) and pI from the MSDB database. c, Probability-based MOWSE (molecular weight search) scores. d, The number of unique peptides identified by MS/MS sequencing, and individual ions scores are all identity or extensive homology (p < 0.05). Click here for file

Additional file 5 **Figure S1.** Results of mitochrondrial voltage-dependent anion-selective channel in the adventitious roots of cassava cultivar SC8 as a representation using LC-ESI-MS/MS. A, protein view and B, peptide view of Mascot search results. Click here for file

Additional file 6 **Table S5.** Details of unique proteins to somatic embryos, shoots, adventitious roots and tuberous roots of cassava SC8, respectively. Click here for file

Additional file 7 **Figure S2.** Proteins on nitrocellulose membranes were detected with Ponceau staining. Proteins transferred from SDS-PAGE gels to nitrocellulose membranes were stained with 5% Ponceau in 1% acetic acid for several minutes, and then washed with 1% acetic acid. A, For Rubisco detection with antiRubisco-polyclonal antibody and B, For α-tubulan detection with anti-α-tubulin-monoclonal antibody. ARs, adventitious roots. Click here for file

Additional file 8 **Figure S3.** Analysis of protein fractions by LabChip GXII Electrophoresis System. A, Protein fraction patterns of cassava cultivar SC8 somatic embryos, plantlets (adventitious roots and shoots) and tuberous roots. The left lane represents molecular mass markers. B, Protein fraction intensity detected by fluorescence analysed with LabChipGX program (Version 1.1.119.0). The arrow presents the molecular weight of one protein fraction. Click here for file
