# Supplementary material for: Proteome characterization of cassava (Manihot esculenta Crantz) somatic embryos, plantlets and tuberous roots
Source: Proteome Sci. 2010 Feb 27;8:10. doi: 10.1186/1477-5956-8-10 (PMC2842255; doi:10.1186/1477-5956-8-10)
Supplement: Additional file 1 — Table S1. Protein identification in cassava cultivars SC8 somatic embryos. a, MSDB accession number. b, Theoretical molecular mass (kDa) and pI from the MSDB database. c, Probability-based MOWSE (molecular weight search) scores. d, The number of unique peptides identified by MS/MS sequencing, and individual ions scores are all identity or extensive homology (p < 0.05). [file 1477-5956-8-10-S1.PDF]

Additional file 1, Table S1

Protein name	Accession no[a]	Theoretical molecular mass (KDa)/pI[b]	Score[c]	Sequence coverage (%)	No. of total peptides matched	No. of unique peptides matched[d]
***Structure* (4)**						
Actin - *Gossypium hirsutum* (Upland cotton)	Q7XZI7_GOSHI	41.701/5.31	161	13	5	3
Alpha tubulin 1 - *Pseudotsuga menziesii* var. menziesii	Q5ME66_PSEMZ	49.610/4.93	99	10	3	2
Alpha-tubulin 4 (Fragment) - *Gossypium hirsutum* (Upland cotton)	Q8H6L8_GOSHI	34.013/5.36	94	20	7	4
Beta-tubulin (Fragment) - *Micromonas pusilla*	Q3T2B6_9CHLO	43.126/5.68	56	19	4	3
***Defense* (3)**						
Pathogenesis-related protein 18 - garden pea	S42650	17.013/5.25	107	10	2	2
Putative NBS-LRR type disease resistance protein (Fragment) - *Pisum sativum* (Garden pea)	Q9SPZ7_PEA	19.387/4.98	62	6	2	1
Peptidyl prolyl cis-trans isomerase (Fragment) - *Arabidopsis thaliana* (Mouse-ear cress)	Q6LAB2_ARATH	6.832/6.51	188	22	2	1
***Inorganic ion transport and metabolism* (3)**						
Calmodulin (CaM) - *Triticum aestivum* (Wheat)	CALM_WHEAT	16.705/4.10	136	22	2	2
Calreticulin - castor bean	T10172	47.493/4.38	82	7	2	1
Voltage-dependent anion channel protein - spinach	T09116	29.610/6.59	85	11	2	1
***Detoxifying and antioxidant* (3)**						
Ascorbate peroxidase APX3- *Manihot esculenta* (Cassava) (Manioc)	Q52QX1_MANES	27.652/5.31	256	27	12	4
Superoxide dismutase (Mn) - Para rubber tree	S39492	25.823/7.10	68	6	1	1
Monodehydroascorbate reductase I - *Pisum sativum* (Garden pea)	Q66PF9_PEA	47.321/5.79	66	4	1	1
***Signal transduction mechanisms* (2)**						
14-3-3 protein - *Manihot esculenta* (Cassava) (Manioc)	Q1AP39_MANES	29.813/4.75	291	37	12	5
Proliferating cell nuclear antigen (clone 4.10) - carrot	S20592	29.218/4.67	52	19	4	1
***Carbohydrate and energy metabolism associated proteins* (26)**						
Alcohol dehydrogenase - *Arabis blepharophylla* (Coast rock-cress)	Q9S700_ARABL	40.968/5.65	232	11	4	2
Alcohol dehydrogenase 1 - garden petunia	DEPJA1	41.547/6.19	52	6	2	2
Alcohol dehydrogenase 2a - upland cotton	S71570	41.048/5.97	168	11	7	3
Cellulase Cel3, membrane-anchored - tomato	T07612	68.512/8.94	49	5	4	1

Cytosolic 3-phosphoglycerate kinase (Fragment) - *Hordeum vulgare* var. distichum (Two-rowed barley)	Q850M2_HORVD	31.302/5.05	67	15	3	2
Cytosolic phosphoglycerate kinase 1- *Populus nigra* (Lombardy poplar)	O82159_POPNI	42.642/5.70	130	12	3	2
Enolase - *Gossypium barbadense* (Egyptian cotton)	Q6WB92_GOSBA	47.702/6.16	560	28	15	7
Fructokinase-like protein - *Solanum tuberosum* (Potato)	Q2PYY9_SOLTU	34.413/5.47	63	4	2	1
Fructose-bisphosphate aldolase, cytosolic - common ice plant	T12416	38.134/6.49	163	19	10	4
Glyceraldehyde-3-phosphate dehydrogenase (phosphorylating) - *Magnolia liliiflora*	DEJMG	36.959/7.10	600	21	22	5
Glyceraldehyde-3-phosphate-dehydrogenase - *Lupinus albus* (White lupin)	Q53I52_LUPAL	32.166/6.8	461	22	12	4
Isocitrate dehydrogenase (Fragment) - *Theobroma cacao* (Cacao) (Cocoa)	Q1HPA5_THECC	10.896/8.74	50	34	2	1
Ketol-acid reductoisomerase (Fragment) - *P. acerifolia* (London plane tree)	Q1M2Z5_PLAAC	18.500/10.47	76	10	1	1
Malate dehydrogenase precursor, mitochondrial - watermelon	DEPUMW	36.178/8.88	104	16	6	3
Malate dehydrogenase, cytosolic - common ice plant	T12433	35.475/6.00	76	11	2	2
NADH dehydrogenase subunit F (Fragment) - *Typha angustifolia* (Narrow leaf cattail)	O47212_TYPAN	77.016/9.13	61	2	3	1
Phosphoglycerate kinase, putative - *A. thaliana* (Mouse-ear cress)	Q8LFV7_ARATH	42.121/5.49	231	27	10	7
Phosphoglycerate mutase, 2,3-bisphosphoglycerate-independent - castor bean	S49647	60.780/5.52	85	9	3	1
Putative pyruvate kinase - *Arabidopsis thaliana* (Mouse-ear cress)	Q8LFE1_ARATH	57.473/5.77	50	7	2	1
Pyruvate kinase - *Glycine max* (Soybean)	Q8L7J4_SOYBN	55.281/7.06	138	9	7	2
Pyruvate kinase-like (Fragment) - *Deschampsia antarctica* (Antarctic hairgrass).	Q8LPV6_DESAN	53.409/6.31	174	9	5	3
Sucrose synthase - *Arabidopsis thaliana* (Mouse-ear cress)	SUS1_ARATH	92.740/5.84	224	14	17	5
F1-ATPase alpha subunit (Fragment) - *Humbertia madagascariensis*	Q5S817_9ASTE	46.153/5.98	65	4	1	1
H+-transporting two-sector ATPase alpha chain - kidney bean mitochondrion	S26979	55.310/6.51	64	5	2	2
H+-transporting two-sector ATPase beta chain, mitochondrial - Para rubber tree	S20504	60.221/5.95	474	25	11	5

Putative ATP synthase beta subunit - *Oryza sativa* (japonica cultivar-group)	Q5N7P8_ORYSA	45.208/5.26	61	5	1	1
DNA and RNA metabolism associated proteins (8)						
3-oxoacyl-[acyl-carrier protein] reductase (Fragment) - *Malus domestica* (Apple) (Malus sylvestris)	Q6V8M2_MALDO	12.427/9.07	51	19	1	1
Phosphoribosylamine-glycine ligase, chloroplast, putative, expressed - *Oryza sativa* (japonica cultivar-group)	Q2QWF3_ORYSA	54.727/5.60	56	2	1	1
Putative DEAD box protein - *Oryza sativa* (japonica cultivar-group)	Q5QLP5_ORYSA	48.291/9.57	55	3	2	1
RNA-binding protein-like - *Oryza sativa* (japonica cultivar-group)	Q5ZDR1_ORYSA	78.156/8.68	65	2	1	1
RNase H, putative (Retrotransposon protein, putative, unclassified) - *Oryza sativa* (japonica cultivar-group)	Q53MZ2_ORYSA	134.767/6.80	59	1	1	1
Gbf1 protein (Fragment) - *Fagus sylvatica* (Beechnut)	Q9FSU2_FAGSY	29.359/6.17	61	4	1	1
MatK (Fragment) - *Dioon mejiae*	Q95B66_9SPER	55.432/9.74	50	2	1	1
Maturase-like protein - *Adesmia volckmannii*	Q9TKT4_9FABA	61.146/8.98	71	3	4	1
DNA binding proteins (3)						
Histone H2B.10 (HTB2) - *Arabidopsis thaliana* (Mouse-ear cress)	H2B10_ARATH	15.592/10.05	107	18	3	2
Histone H4 - garden pea	HSPM4	11.402/11.48	74	33	4	2
Putative DNA-binding protein GBP16 - *Oryza sativa* (japonica cultivar-group)	Q5W6H1_ORYSA	43.170/6.62	72	4	1	1
Amino acid metabolism (7)						
Aspartate aminotransferase P1 - *Lupinus angustifolius* (Narrow-leaved blue lupin)	Q40107_LUPAN	45.827/8.36	111	10	6	3
Cysteine proteinase - *Arabidopsis thaliana* (Mouse-ear cress)	Q9LT78_ARATH	49.276/5.60	47	2	1	1
Glutamine synthetase - *Lactuca sativa*	CAA42689	39.446/5.24	78	8	2	2
Methionine synthase (Fragment) - Coffea arabica (Coffee)	Q9M619_COFAR	24.430/5.69	112	13	2	2
Putative alanine aminotransferase (Fragment) - *A. thaliana* (Mouse-ear cress)	Q94C83_ARATH	58.415/5.62	72	3	1	1
S-adenosylmethionine synthetase (Fragment) - *Oryza rufipogon* (Wild rice).	Q1H960_ORYRU	42.564/5.74	57	3	1	1
Transaldolase-like - *Solanum tuberosum* (Potato)	Q2XTB7_SOLTU	47.883/5.95	74	9	2	2
Protein biosynthesis (31)						
40S ribosomal protein S10-like - *Solanum tuberosum* (Potato)	Q2XPV4_SOLTU	19.830/9.79	48	8	1	1

40S ribosomal protein S3a - *Catharanthus roseus* (Madagascar periwinkle)	Q1SLE0_MEDTR	29.503/9.77	57	11	1	1
40S ribosomal protein S3a-like – *S. tuberosum* (Potato)	Q2VCH9_SOLTU	29.688/9.76	59	4	1	1
40S ribosomal protein S5 - *Oryza sativa* (japonica cultivar-group)	Q2R4A1_ORYSA	22.193/9.72	57	6	1	1
60S ribosomal protein L12 - *Capsicum annuum* (Bell pepper)	Q6RJY1_CAPAN	17.704/8.81	67	16	2	2
Elongation factor 1-alpha - *Zea mays* (Maize)	O50018_MAIZE	49.259/9.19	136	17	7	2
Elongation factor Tu - *Medicago truncatula* (Barrel medic)	Q1S824_MEDTR	94.123/5.91	146	2	2	1
Elongation factor-1 alpha 2 - *Lilium longiflorum* (Trumpet lily)	Q9SPA2_LILLO	49.428/9.15	55	11	4	2
Eukaryotic initiation factor 4A - *Pennisetum americanum* (Pearl millet)	Q4U474_PENAM	46.992/5.36	88	15	7	4
Initiation factor eIF4A-15 - *Helianthus annuus* (Common sunflower)	Q6T8C6_HELAN	46.580/5.29	81	11	3	2
Mitochondrial ribosomal protein L5 - *Medicago truncatula* (Barrel medic)	Q1S6P8_MEDTR	20.672/9.97	60	7	1	1
Peptidase, cysteine peptidase active site; Ribosomal protein L30 - *Medicago truncatula* (Barrel medic)	Q2HVI3_MEDTR	28.486/9.90	55	5	1	1
Proteasome subunit alpha type 2, putative - *Musa acuminata* (Banana)	Q1EP82_MUSAC	6.910/5.54	75	31	1	1
Putative 40S Ribosomal protein - *O. sativa* (Rice)	Q94HF0_ORYSA	33.122/4.86	58	10	2	2
Putative 40S ribosomal protein S2 - *O. sativa* (japonica cultivar-group)	Q6ZIW7_ORYSA	30.090/10.20	138	5	2	1
Putative 40S ribosomal protein S6 - *O. sativa* (japonica cultivar-group)	Q8LH97_ORYSA	28.455/10.66	87	12	2	1
Putative 40S ribosomal protein S8-like protein - *Solanum tuberosum* (Potato)	Q2XPV9_SOLTU	24.969/10.40	132	19	3	2
Putative 60S ribosomal protein L24 - *O. sativa* (japonica cultivar-group)	Q8L3Y6_ORYSA	18.202/10.72	56	7	1	1
Putative 60S ribosomal protein L9 - *O. sativa* (japonica cultivar-group)	Q6ZFJ5_ORYSA	20.655/9.65	49	29	3	1
Putative beta 3 proteasome subunit (Fragment) - *Nicotiana tabacum* (Common tobacco)	Q93X33_TOBAC	19.832/5.08	57	17	2	1
Putative Csa-19 - *Oryza sativa* (japonica cultivar-group)	Q5QL84_ORYSA	15.090/10.15	51	10	1	1
Putative elongation factor 1-a - *Arabidopsis thaliana* (Mouse-ear cress)	Q8W4H7_ARATH	49.531/9.19	52	4	2	1
Ribosomal protein L12 (Fragment) - Cichorium intybus (Chicory)	Q9ZSL1_CICIN	15.166/9.88	101	21	2	2
Ribosomal protein L29 - *Medicago truncatula* (Barrel medic)	Q1S449_MEDTR	19.434/10.69	72	7	2	1
ribosomal protein L7a, cytosolic - *Arabidopsis thaliana*	T00423	29.111/10.14	62	5	1	1
Ribosomal protein S3 - Norway spruce chloroplast	T11807	25.380/9.62	68	6	1	1
Ribosomal protein S5, bacterial and organelle form - *Medicago truncatula* (Barrel medic)	Q1SLE0_MEDTR	30.431/10.32	72	4	2	1

Ribosomal protein small subunit 28 - *Helianthus annuus* (Common sunflower)	Q8LK53_HELAN	7.503/11.16	59	18	1	1
Translation elongation factor 1A-1 - *Gossypium hirsutum* (Upland cotton)	Q3LUM6_GOSHI	49.196/9.15	141	6	3	2
Translation factor - *Medicago truncatula* (Barrel medic)	Q1S825_MEDTR	94.081/5.8	58	4	4	2
Ubiquitin/s27a 40s ribosomal protein - *Nicotiana benthamiana*	Q5I6U1_NICBE	17.693/9.77	66	10	3	1
Chaperones (9)						
Chaperonin Cpn60/TCP-1 - *Medicago truncatula* (Barrel medic).	Q1SDY8_MEDTR	31.483/6.54	58	5	1	1
Heat shock protein 70 - *Cucumis sativus* (Cucumber)	Q9M4E6_CUCSA	70.784/5.29	403	18	10	4
Heat shock protein 90 (Fragment) - *Triticum aestivum* (Wheat)	Q2L3T8_WHEAT	75.587/5.02	86	4	2	2
Heat-shock inducible Hsp70- *Volvox carteri* f. nagariensis.	Q3I5Q5_VOLCA	71.010/5.19	157	4	4	2
High molecular weight heat shock protein - *Malus domestica* (Apple) (Malus sylvestris)	Q9M6R1_MALDO	71.171/5.17	301	13	10	3
Molecular chaperone Hsp90-1 - *Nicotiana benthamiana*	Q6UJX6_NICBE	80.055/4.94	100	11	8	3
Putative dnaK-type molecular chaperone - *Oryza sativa* (japonica cultivar-group)	Q6Z7L1_ORYSA	72.853/5.49	83	2	1	1
Putative HEAT SHOCK PROTEIN 81-2 (Fragment) - *Trifolium pratense* (Red clover)	Q2PEX3_TRIPR	55.394/5.16	106	16	7	3
T-complex protein 1, alpha subunit (Fragment) - *Medicago truncatula* (Barrel medic)	Q1RV32_MEDTR	55.886/6.45	89	7	2	1
Sorting and translocation (1)						
Leucine Rich Repeat, putative - *Oryza sativa* (japonica cultivar-group)	Q53QA7_ORYSA	115.553/7.34	50	1	1	1
Transport (7)						
ADP, ATP carrier protein CANT1 - upland cotton	T09709	42.067/9.88	192	11	6	3
ADP-ribosylation factor - *Zea mays* (Maize)	ARF_MAIZE	20.517/6.36	143	16	3	2
Cytochrome P450-like protein - *Arabidopsis thaliana* (Mouse-ear cress)	Q9LHR3_ARATH	41.635/8.08	48	5	2	1
Cytokinin binding protein CBP57 - *Nicotiana sylvestris* (Wood tobacco)	Q42939_NICSY	49.227/6.10	108	10	5	1
Putative chloroplast thiazole biosynthetic protein - *Nicotiana tabacum* (Common tobacco)	Q84QE4_TOBAC	37.947/5.76	97	10	2	1
Thiamine biosynthetic enzyme - *Picrorhiza kurrooa*	Q5G1J2_9LAMI	37.459/5.03	88	7	3	1

Thiazole biosynthetic enzyme (Fragments) - *Populus euphratica* (Euphrates poplar)	THI4_POPEU	5.122/4.77	122	43	1	1
Function unknown proteins (5)						
AF255338 NID - *Glycine max*	AAF70292	25.964/4.70	68	12	2	2
Arabidopsis thaliana genomic DNA, chromosome 5, P1 clone:MEE6 - *Arabidopsis thaliana* (Mouse-ear cress)	Q9FLL1_ARATH	66.684/5.55	66	2	4	1
Hypothetical protein - *Citrus paradisi* (Grapefruit)	O04428_CITPA	32.623/5.46	82	9	2	2
Hypothetical protein - *Solanum tuberosum* (Potato)	Q38M64_SOLTU	32.511/9.37	69	13	3	1
Hypothetical protein - *Solanum tuberosum* (Potato)	Q38M76_SOLTU	21.963/9.59	160	16	2	2
**The total protein number**			**112**			
