# Supplementary material for: Proteome characterization of cassava (Manihot esculenta Crantz) somatic embryos, plantlets and tuberous roots
Source: Proteome Sci. 2010 Feb 27;8:10. doi: 10.1186/1477-5956-8-10 (PMC2842255; doi:10.1186/1477-5956-8-10)
Supplement: Additional file 2 — Table S2. Protein identification in cassava cultivars SC8 shoots. a, MSDB accession number. b, Theoretical molecular mass (kDa) and pI from the MSDB database. c, Probability-based MOWSE (molecular weight search) scores. d, The number of unique peptides identified by MS/MS sequencing, and individual ions scores are all identity or extensive homology (p < 0.05). [file 1477-5956-8-10-S2.PDF]

Additional file 2, Table S2

Protein name	Accession no[a]	Theoretical molecular mass (KDa)/pI[b]	Score[c]	Sequence coverage (%)	No. of total peptides matched	No. of unique peptides matched[d]
Structure (5)						
Actin - *Gossypium hirsutum* (Upland cotton)	Q7XZI7_GOSHI	41.701/5.31	399	28	11	5
Alpha-tubulin 4 (Fragment) - *Gossypium hirsutum* (Upland cotton)	Q8H6L8_GOSHI	34.013/5.36	309	27	9	4
Putative tubulin alpha-2/alpha-4 chain - *Brassica napus* (Rape)	Q6UBL2_BRANA	49.405/4.91	157	26	10	3
Beta-tubulin (Fragment)- *Zinnia elegans* (Zinnia)	Q9STC9_ZINEL	49.359/4.68	119	17	6	5
Putative gamma-tubulin complex component 4 - *Oryza sativa* (japonica cultivar-group)	Q60F24_ORYSA	85.891/8.45	66	3	2	1
Defense (2)						
Peptidyl prolyl cis-trans isomerase (Fragment) - *Arabidopsis thaliana* (Mouse-ear cress)	Q6LAB2_ARATH	6.832/6.51	151	22	3	1
Peptidylprolyl isomerase Cyp - kidney bean	S54833	18.148/8.36	152	15	3	1
Inorganic ion transport and metabolism (1)						
Calmodulin (CaM) - *Triticum aestivum* (Wheat)	CALM_WHEAT	16.705/4.10	64	10	2	1
Detoxifying and antioxidant (7)						
Ascorbate peroxidase APX3 - *Manihot esculenta* (Cassava) (Manioc)	Q52QX1_MANES	27.652/5.31	155	20	8	2
Catalase CAT1 - *Manihot esculenta* (Cassava) (Manioc)	Q9SW99_MANES	57.137/6.87	61	11	4	3
Ferredoxin: nadp+ reductase mutant Y308S, chain A - garden pea	1QFYA	34.699/6.54	93	9	3	1
Geranylgeranyl reductase - *Medicago truncatula* (Barrel medic)	Q56GA3_MEDTR	51.122/9.01	65	4	2	1
Glycolate oxidase - *Mesembryanthemum crystallinum* (Common ice plant)	P93260_MESCR	40.587/9.02	79	14	4	3
Monodehydroascorbate reductase I - *Pisum sativum* (Garden pea)	Q66PF9_PEA	47.321/5.79	71	4	1	1
Protochlorophyllide reductase precursor - cucumber	JC4146	43.047/9.05	47	4	2	1
Signal transductio mechanisms (3)						
14-3-3 protein - *Manihot esculenta* (Cassava) (Manioc)	Q1AP39_MANES	29.813/4.75	68	14	4	2
Putative pollen signalling protein with adenylyl cyclase activity - *Oryza sativa* (japonica cultivar-group)	Q6K3A0_ORYSA	122.116/5.66	50	1	1	1

Receptor kinase TRKc - *Oryza sativa* (indica cultivar-group)	Q2EZ09_ORYSA	121.482/6.07	48	0	1	1
Photosynthesis related proteins (24)						
Chlorophyll a/b binding protein precursor - *Cicer arietinum* (Chickpea) (Garbanzo)	Q9ZP08_CICAR	28.300/5.47	71	21	5	2
Chloroplast chlorophyll A/B binding protein (Fragment) - *Manihot esculenta* (Cassava) (Manioc)	Q5PYQ1_MANES	26.253/5.44	54	5	3	1
Chloroplast latex aldolase-like protein (Fragment) - *Manihot esculenta* (Cassava) (Manioc)	Q5PYQ2_MANES	33.788/6.22	146	27	9	3
Cytochrome f soluble domain, chain B - turnip	2PCFB	27.272/6.23	70	11	2	1
Light-harvesting chlorophyll-a/b binding protein Lhcb1 - *Pisum sativum* (Garden pea)	Q5I8X3_PEA	28.375/5.48	74	4	2	1
P700 apoprotein A2 of photosystem I - *Scenedesmus obliquus*	Q1KVS5_SCEOB	81.850/6.47	50	1	1	1
Photosystem I protein PsaD - *Medicago truncatula* (Barrel medic)	Q1SDE6_MEDTR	23.034/9.58	114	13	9	2
Photosystem I reaction center subunit III - *Phaseolus aureus* (Mung bean) (Vigna radiata)	Q9XQB4_PHAAU	24.320/9.66	59	5	1	1
Photosystem I reaction centre subunit IV/PsaE - *Medicago truncatula* (Barrel medic)	Q1SA60_MEDTR	14.956/9.52	68	14	2	1
Photosystem II CP43 chlorophyll apoprotein - *Helianthus annuus* (Common sunflower)	Q1KXW3_HELAN	51.851/6.68	192	9	6	3
Photosystem II CP47 chlorophyll apoprotein - *Gossypium hirsutum* (Upland cotton)	Q2L932_GOSHI	56.063/6.27	271	12	8	4
Photosystem II D2 protein (Photosystem Q(A) protein) (PSII D2 protein) - *Arabidopsis thaliana* (Mouse-ear cress)	PSBD_ARATH	39.391/5.46	164	11	3	2
Photosystem II oxygen-evolving complex protein 1 precursor - tomato	T06368	34.926/5.91	347	23	15	5
Photosystem II oxygen-evolving complex protein 2 - rice (fragment)	PS0187	4.038/9.53	81	35	2	1
Photosystem Q(B) protein - *Arabidopsis thaliana* (Mouse-ear cress)	PSBA_ARATH	38.780/5.12	60	3	2	1
Plastoquinol-plastocyanin reductase cytochrome f precursor - common tobacco chloroplast	CFNT	35.224/9.12	60	5	2	1
Putative chloroplast photosystem I subunit III (Fragment) - *Populus canadensis* (Carolina poplar)	Q2EHP8_POPCA	6.889/6.34	61	24	1	1

Putative oxygen-evolving enhancer protein 1 (OEE1) (Fragments) - *Pinus strobus* (Eastern white pine)	PSBO_PINST	13.644/4.46	66	35	6	3
Putative photosystem I reaction centre PSI-D subunit precursor - *Solanum tuberosum* (Potato)	Q70PN9_SOLTU	22.792/9.63	63	22	5	2
Ribulose 1,5-bisphosphate carboxylase small chain - *Manihot esculenta* (Cassava) (Manioc)	Q9T4H0_MANES	20.392/8.33	225	32	21	6
Ribulose-1,5-bisphosphate carboxylase/oxygenase activase 1 - *Gossypium hirsutum* (Upland cotton)	Q9AXG1_GOSHI	47.958/5.54	322	17	19	4
Ribulose-1,5-bisphosphate carboxylase/oxygenase activase 2 (Fragment) - *Gossypium hirsutum* (Upland cotton)	Q9AXG0_GOSHI	48.324/5.06	166	16	10	4
Ribulose-1,5-bisphosphate carboxylase/oxygenase large subunit (Fragment) - *Opilia amentacea*	Q32726_9MAGN	52.316/5.83	329	13	14	3
Ribulose-bisphosphate carboxylase (Fragment) - *Leptonychia pallida*	Q9XQI4_9ROSI	51.505/5.96	599	26	38	6
Carbohydrate and energy metabolism associated proteins (25)						
(S)-acetone-cyanohydrin lyase - *Manihot esculenta* (Cassava) (Manioc)	HNL_MANES	29.222/6.00	277	56	26	10
beta-glucosidase - cassava	S23940	61.334/5.52	257	13	16	6
Enolase - *Gossypium barbadense* (Egyptian cotton)	Q6WB92_GOSBA	47.689/5.31	104	13	5	2
Enoyl ACP reductase - *Olea europaea* subsp. europaea	Q8GVC8_OLEEU	41.855/8.20	71	6	2	1
Fructose-bisphosphate aldolase, cytosolic - common ice plant	T12416	38.134/6.49	98	12	6	2
Glyceraldehyde-3-phosphate dehydrogenase (Fragment) - *Helianthus annuus* (Common sunflower)	Q15CX3_HELAN	23.996/5.64	258	31	15	5
glyceraldehyde-3-phosphate dehydrogenase (NADP) (phosphorylating) A precursor, chloroplast - garden pea	DEPMNA	43.312/8.80	269	14	11	3
Glycine dehydrogenase P protein - *Oryza sativa* (japonica cultivar-group)	Q6V9T1_ORYSA	111.356/6.51	76	3	2	1
Ketol-acid reductoisomerase (Fragment) - *Platanus acerifolia* (London plane tree)	Q1M2Z5_PLAAC	18.500/10.47	87	10	1	1
Malate dehydrogenase, cytosolic - common ice plant	T12433	35.475/6.00	135	17	9	3
phosphoglycerate kinase precursor, chloroplast - common tobacco	T03660	50.146/8.48	187	16	8	5
Putative dehydrogenase (Dehydrogenase, putative) - *Oryza sativa* (japonica cultivar-group)	Q8W3D9_ORYSA	42.739/9.00	84	7	3	1

Sucrose synthase (Fragment) - *Manihot esculenta* (Cassava) (Manioc)	Q5PYQ4_MANES	31.489/5.44	84	8	2	1
Transketolase precursor, chloroplast - spinach	T09015	80.231/6.20	111	4	4	1
Transketolase, C-terminal-like - *Medicago truncatula* (Barrel medic)	Q1T6A8_MEDTR	79.800/6.44	72	2	1	1
AAA ATPase, central region; Homeodomain-like - *Medicago truncatula* (Barrel medic)	Q1SZ48_MEDTR	52.251/6.10	397	22	23	4
ATP synthase beta subunit (Fragment) - *Primula palinuri*	Q9GER5_9ERIC	47.638/4.78	317	13	10	5
ATP synthase subunit alpha - *Nicotiana tabacum* (Common tobacco)	ATPA_TOBAC	55.420/5.14	62	9	2	2
AtpB (Fragment) - *Ceratophyllum submersum* (Tropical hornwort) (ATP synthase, beta subunit)	Q95CV0_CERSB	52.232/5.19	481	43	35	11
ATP-dependent Clp protease, ATP-binding subunit - *Arabidopsis thaliana* (Mouse-ear cress)	Q9FI56_ARATH	103.388/6.36	120	5	3	2
F1-ATPase alpha subunit (Fragment) - *Plantago subspathulata*	Q5S7Z0_9LAMI	45.553/7.78	62	11	3	1
H+-transporting two-sector ATPase alpha chain - garden pea chloroplast	PWPMA	54.492/5.75	120	8	3	3
H+-transporting two-sector ATPase beta chain, mitochondrial - Para rubber tree	S20504	60.221/5.95	241	20	10	4
H+-transporting two-sector ATPase epsilon chain - spinach chloroplast	PWSPE	14.691/6.59	67	15	2	1
H+-transporting two-sector ATPase gamma chain precursor, chloroplast - spinach	PWSPG	40.049/5.95	69	7	2	1
DNA and RNA metabolism associated proteins (3)						
Putative DEAD box protein - *Oryza sativa* (japonica cultivar-group)	Q5QLP5_ORYSA	48.291/9.57	76	6	2	1
Maturase K - *Peperomia graveolens*	Q2F7X8_9MAGN	60.846/9.45	48	2	1	1
Maturase-like protein - *Adesmia volckmannii*	Q9TKT4_9FABA	61.146/8.98	57	3	2	1
DNA binding protein (2)						
Histone H2B.10 (HTB2) - Arabidopsis thaliana (Mouse-ear cress)	H2B10_ARATH	15.592/10.05	152	18	9	2
Histone H4 - garden pea	HSPM4	11.402/11.48	198	33	5	3
Amino acid metabolism (4)						

5-methyltetrahydropteroyltriglutamate-homocysteine S-methyltransferase - *Madagascar periwinkle*	S57636	84.804/6.10	54	2	1	1
Glutamate 1-semialdehyde aminotransferase - *Brassica napus* (Rape)	Q84TK5_BRANA	50.289/6.22	61	6	2	1
Glutamine synthetase - *Canavalia lineata*	Q9SEX6_CANLI	47.385/6.28	70	3	3	1
S-adenosylmethionine synthetase (Fragment) - *Oryza rufipogon* (Wild rice)	Q1H960_ORYRU	42.564/5.74	142	10	3	3
Protein biosynthesis (14)						
50S ribosomal protein L12, chloroplast (Fragment) - *Populus euphratica* (Euphrates poplar)	RK12_POPEU	1.488/4.25	82	100	1	1
60S ribosomal protein L12 - *Capsicum annuum* (Bell pepper)	Q6RJY1_CAPAN	17.704/8.81	55	16	2	1
Elongation factor 1, gamma chain - *Medicago truncatula* (Barrel medic)	Q1SL16_MEDTR	47.694/6.43	106	7	2	2
Elongation factor 1-alpha - *Zea mays* (Maize)	O50018_MAIZE	49.259/9.19	93	14	12	3
Eukaryotic initiation factor 4A - *Pennisetum americanum* (Pearl millet)	Q4U474_PENAM	46.992/5.36	142	9	5	3
Mitochondrial ribosomal protein L5 - *Medicago truncatula* (Barrel medic)	Q1S6P8_MEDTR	20.672/9.97	72	7	1	1
Peptidase, cysteine peptidase active site; Ribosomal protein L30 - *Medicago truncatula* (Barrel medic)	Q2HVI3_MEDTR	28.486/9.90	52	5	1	1
Putative 40S ribosomal protein S8-like protein - *Solanum tuberosum* (Potato)	Q2XPV9_SOLTU	24.969/10.40	85	19	3	3
Putative elongation factor 2 - *Oryza sativa* (japonica cultivar-group)	Q6H4L2_ORYSA	93.961/5.85	89	4	2	1
Ribosomal protein L12 (Fragment) - *Cichorium intybus* (Chicory)	Q9ZSL1_CICIN	15.166/9.88	67	21	2	1
Ribosomal protein L29 - *Medicago truncatula* (Barrel medic)	Q1S449_MEDTR	19.434/10.69	60	7	1	1
Ribosomal protein S19e - *Medicago truncatula* (Barrel medic)	Q1S1G5_MEDTR	15.946/10.05	66	9	1	1
Ribosome-associated protein p40-like - *Solanum tuberosum* (Potato)	Q38M60_SOLTU	30.198/5.09	74	8	3	1
Translation elongation factor EF-Tu precursor, chloroplast - garden pea	T06821	53.017/6.62	130	5	5	2
Chaperones (9)						
Chaperonin 21 precursor - *Lycopersicon esculentum* (Tomato)	Q9M5A8_LYCES	26.546/6.85	62	4	1	1

Chaperonin groEL - castor bean (fragment)	HHCSBA	52.347/4.77	317	25	15	5
Chloroplast envelope membrane 70 kDa heat shock-related protein - *Spinacia oleracea* (Spinach)	HSP7E_SPIOL	71.555/5.35	146	14	8	5
Heat shock protein 70 - *Cucumis sativus* (Cucumber)	Q9M4E7_CUCSA	71.444/5.07	283	19	16	9
Heat shock protein 70a - *Dunaliella salina*	Q8RY44_DUNSA	70.964/5.31	75	4	2	2
Heat shock protein, 70K, chloroplast - cucumber	T10248	75.366/5.15	188	12	9	5
Molecular chaperone Hsp90-1 - *Nicotiana benthamiana*	Q6UJX6_NICBE	80.055/4.94	243	11	10	2
Probable chaperonin 60 beta chain - garden pea chloroplast	T06412	62.945/5.85	258	21	15	5
Putative chaperonin 60 beta - *Oryza sativa* (japonica cultivar-group)	Q9LWT6_ORYSA	64.046/5.60	145	12	9	3
Transport (3)						
Concanavalin A-like lectin/glucanase - *Medicago truncatula* (Barrel medic)	Q1S0P2_MEDTR	48.424/4.41	58	3	1	1
ATP-binding cassette transporter AtABCA1 - *Arabidopsis thaliana* (Mouse-ear cress)	Q8W010_ARATH	209.091/6.45	49	0	1	1
Cyclophilin - *Ricinus communis* (Castor bean)	Q8VX73_RICCO	18.142/8.94	98	15	2	1
Function unknown proteins (8)						
4D11_26 - *Brassica rapa* subsp. pekinensis (Chinese cabbage)	Q4ABW1_BRARP	41.726/11.04	57	4	1	1
AF255338 NID - *Glycine max*	AAF70292	25.964/4.70	74	6	1	1
AGSUS1 NID - *Alnus glutinosa*	CAA63122	91.573/6.35	67	2	2	1
Arabidopsis thaliana genomic DNA, chromosome 5, P1 clone:MEE6 - Arabidopsis thaliana (Mouse-ear cress)	Q9FLL1_ARATH	66.684/5.55	77	2	4	1
DQ347958 NID - *Solanum bulbocastanum*	ABC56198	55.377/5.14	116	17	7	5
Protein At3g24760 - Arabidopsis thaliana (Mouse-ear cress)	Q3EB08_ARATH	42.645/5.50	54	5	3	1
Putative RUSH-1alpha - *Oryza sativa* (japonica cultivar-group)	Q8GSA1_ORYSA	91.274/8.98	48	1	1	1
ZMB3TUB NID - *Zea mays*	CAA52718	49.778/4.79	71	16	5	3
**The total protein number**			**110**			
