# Supplementary material for: Proteome characterization of cassava (Manihot esculenta Crantz) somatic embryos, plantlets and tuberous roots
Source: Proteome Sci. 2010 Feb 27;8:10. doi: 10.1186/1477-5956-8-10 (PMC2842255; doi:10.1186/1477-5956-8-10)
Supplement: Additional file 3 — Table S3. Protein identification in cassava cultivars SC8 adventitious roots. a, MSDB accession number. b, Theoretical molecular mass (kDa) and pI from the MSDB database. c, Probability-based MOWSE (molecular weight search) scores. d, The number of unique peptides identified by MS/MS sequencing, and individual ions scores are all identity or extensive homology (p < 0.05). [file 1477-5956-8-10-S3.PDF]

Additional file 3, Table S3

Protein name	Accession no[a]	Theoretical molecular mass (KDa)/pI[b]	Score[c]	Sequence coverage (%)	No. of total peptides matched	No. of unique peptides matched[d]
Structure (5)						
Actin - *Gossypium hirsutum* (Upland cotton)	Q7XZI7_GOSHI	41.701/5.31	178	29	15	6
Actin/actin-like - *Medicago truncatula* (Barrel medic)	Q1SQB5_MEDTR	42.286/5.39	83	8	2	2
Alpha-tubulin (Fragment) - *Striga asiatica*	Q1PGA4_9LAMI	22.247/5.25	54	7	1	1
Alpha-tubulin 4 (Fragment) - *Gossypium hirsutum* (Upland cotton)	Q8H6L8_GOSHI	34.013/5.36	133	26	7	4
Tubulin beta chain - *Chlamydomonas reinhardtii*	UBKM	49.587/4.82	112	10	6	3
Defense (6)						
Hemoglobin - *Trema virgata*	Q9SC02_9ROSA	18.140/8.59	121	22	6	2
NBS-LRR type resistance protein (Fragment) - *Cucumis melo* (Muskmelon)	Q588W6_CUCME	11.974/9.25	50	11	2	1
Non-symbiotic hemoglobin protein - *Gossypium hirsutum* (Upland cotton)	Q4VIX3_GOSHI	18.372/8.77	127	14	3	2
Probable resistance protein - *Arabidopsis thaliana*	A71438	101.953/8.37	50	1	1	1
Putative cold-induced protein - *Oryza sativa* (Rice)	Q8S9G2_ORYSA	23.999/10.91	56	5	1	1
Putative NBS-LRR type disease resistance protein (Fragment) - *Pisum sativum* (Garden pea)	Q9SPZ7_PEA	19.387/4.98	52	6	1	1
Inorganic ion transport and metabolism (5)						
Calmodulin (CaM) - *Triticum aestivum* (Wheat)	CALM_WHEAT	16.705/4.10	69	10	4	1
Mitochrondrial voltage-dependent anion-selective channel - *Phaseolus coccineus* (Scarlet runner bean)	Q4PKP6_PHACN	29.710/8.56	77	7	1	1
Outer plastidial membrane protein porin (Voltage-dependent anion- selective channel protein) (VDAC) - *Pisum sativum* (Garden pea).	VDAC_PEA	29.448/9.11	57	9	1	1
voltage-dependent anion channel protein - spinach	T09116	29.610/6.59	136	11	4	1
Ferritin heavy chain precursor - kidney bean	FRFBH	28.286/5.64	57	5	1	1
Detoxifying and antioxidant (11)						
Ascorbate peroxidase APX3 - *Manihot esculenta* (Cassava) (Manioc)	Q52QX1_MANES	27.652/5.31	658	31	33	5

Catalase CAT1 - *Manihot esculenta* (Cassava) (Manioc)	Q9SW99_MANES	57.137/6.87	106	20	10	6
Germin-like protein 2 (Fragment) - *Picea abies* (Picea excelsa)	Q4VSU6_PICAB	17.892/9.11	120	10	7	1
Glutathione peroxidase - *Medicago truncatula* (Barrel medic)	Q1RY57_MEDTR	30.235/9.39	50	8	2	1
Isoflavone reductase related protein - *Pyrus communis* (Pear)	O81355_PYRCO	33.802/6.02	51	3	1	1
Malic oxidoreductase - *Medicago truncatula* (Barrel medic)	Q1SRX6_MEDTR	65.296/5.95	85	6	6	3
Monodehydroascorbate reductase - *Mesembryanthemum crystallinum* (Common ice plant)	Q93YG1_MESCR	51.716/6.38	76	5	2	1
Peroxidase (Fragment) - *Manihot esculenta* (Cassava) (Manioc)	Q94IP4_MANES	26.150/8.37	146	19	8	4
'putative 1,4-benzoquinone reductase' - *Oryza sativa* (japonica cultivar-group)	Q60EJ3_ORYSA	21.616/6.30	68	7	1	1
Putative peroxidase (Class III peroxidase 33 precursor) - *Oryza sativa* (japonica cultivar-group).	Q8LMR7_ORYSA	32.215/5.02	60	4	2	1
Superoxide dismutase (Mn) - Para rubber tree	S39492	25.823/7.10	97	11	4	3
Signal transduction mechanisms (4)						
ADP-ribosylation factor - *Zea mays* (Maize)	ARF_MAIZE	20.517/6.36	106	16	2	1
Cytokinin binding protein CBP57 - *Nicotiana sylvestris* (Wood tobacco)	Q42939_NICSY	49.227/6.10	85	16	5	2
GTP-binding protein SAR1A, putative, expressed - *Oryza sativa* (japonica cultivar-group)	Q2QNM5_ORYSA	22.015/6.91	72	7	1	1
Putative sister-chromatide cohesion protein - *Arabidopsis thaliana* (Mouse-ear cress)	Q8LFH0_ARATH	125.730/5.24	50	2	2	1
Photosynthesis related proteins (6)						
Chlorophyll a/b-binding protein - common tobacco	S14650	28.317/5.27	49	13	1	1
Light-harvesting chlorophyll-a/b binding protein Lhcb1 - *Pisum sativum* (Garden pea)	Q5I8X3_PEA	28.375/5.48	73	4	2	1
Oxygen evolving enhancer protein 1 precursor - *Bruguiera gymnorhiza*	Q9LRC4_9ROSI	35.116/6.48	94	19	6	3
Photosystem II chlorophyll a-binding protein psbB - maize chloroplast	QJZMBB	56.070/6.06	49	2	1	1
Ribulose 1,5-bisphosphate carboxylase small chain - *Manihot esculenta* (Cassava) (Manioc)	Q9T4H0_MANES	20.392/8.33	132	27	8	5
Ribulose-1,5-bisphosphate carboxylase/oxygenase large subunit (Fragment) - *Opilia amentacea*	Q3MKL9_9CARY	50.506/6.11	250	9	14	2

***Carbohydrate and energy metabolism associated proteins* (38)**						
Acetyl-coenzyme A carboxylase - *Alopecurus myosuroides* (Black grass)	Q8LRK2_ALOMY	256.270/5.74	50	1	2	1
Aconitate hydratase - cucurbit	T10101	97.943/5.74	61	8	6	4
Alcohol dehydrogenase (Fragment) - *Miscanthus sinensis* var. sinensis	Q84LZ7_MISSI	32.968/6.28	53	6	1	1
Alcohol dehydrogenase 2a - upland cotton	S71570	41.048/5.97	103	11	5	2
Aldolase (Fragment) - *Triticum aestivum* (Wheat)	Q7X9K7_WHEAT	23.183/8.90	50	5	1	1
Alpha-hydroxynitrile lyase - *Manihot esculenta* (Cassava) (Manioc)	O49897_MANES	29.201/5.23	85	17	3	2
Enolase - *Gossypium barbadense* (Egyptian cotton)	Q6WB92_GOSBA	47.702/6.16	428	29	17	6
Fructose-bisphosphate aldolase, cytosolic - common ice plant	T12416	38.134/6.49	123	19	12	4
GDP-mannose-3,5-epimerase - *Arabidopsis thaliana* (Mouse-ear cress)	CAD70055	42.731/5.85	69	7	2	2
Glyceraldehyde-3-phosphate dehydrogenase (phosphorylating) - *Magnolia liliiflora*	DEJMG	36.959/7.10	377	21	16	4
Glyceraldehyde-3-phosphate-dehydrogenase - *Lupinus albus* (White lupin)	Q53I52_LUPAL	32.166/6.80	451	25	19	5
Malate dehydrogenase, cytosolic - common ice plant	T12433	35.475/6.00	239	25	11	4
Mutator-like transposase [imported] - *Arabidopsis thaliana*	D84484	61.819/9.50	48	2	1	1
Phosphoenolpyruvate carboxylase - *Lotus japonicus*	Q8H946_LOTJA	110.304/5.76	59	6	4	2
phosphogluconate dehydrogenase (decarboxylating) - soybean	T05363	56.338/5.55	61	4	2	2
Phosphoglycerate kinase, putative - *Arabidopsis thaliana* (Mouse-ear cress)	Q8LFV7_ARATH	42.121/5.49	181	18	9	5
Phosphoribulokinase precursor - *Chlamydomonas reinhardtii*	T08167	41.865/8.11	53	5	4	1
Putative 3-dehydroquinate dehydratase/shikimate dehydrogenase - *Fagus sylvatica* (Beechnut)	Q30D00_FAGSY	58.249/6.69	49	1	1	1
Putative cinnamyl alcohol dehydrogenase - *Oryza sativa* (japonica cultivar-group)	Q6ERW9_ORYSA	37.736/6.12	48	4	1	1
Putative mitochondrial NAD-dependent malate dehydrogenase - *Solanum tuberosum* (Potato)	Q8L5C8_SOLTU	36.144/8.48	68	11	3	1
Putative NAD synthetase - *Oryza sativa* (japonica cultivar-group)	Q8LI89_ORYSA	82.025/6.12	66	4	1	1
Pyruvate kinase - *Glycine max* (Soybean)	Q8L7J5_SOYBN	55.282/7.89	63	9	4	2

Pyruvate kinase-like protein - *Arabidopsis thaliana*	T47556	55.916/6.81	131	12	5	1
Succinate dehydrogenase flavoprotein alpha chain [imported] - *Arabidopsis thaliana*	T51815	69.612/5.86	73	8	3	3
Sucrose synthase (Fragment) - *Manihot esculenta* (Cassava) (Manioc)	Q5PYQ4_MANES	31.489/5.44	280	41	16	6
Transaldolase-like- *Solanum tuberosum* (Potato)	Q2XTB7_SOLTU	47.883/5.95	54	9	2	2
Transketolase precursor, chloroplast - spinach	T09015	80.231/6.20	189	4	8	1
Triosphosphate isomerase-like protein - *Solanum tuberosum* (Potato)	Q3HRV9_SOLTU	27.694/5.90	49	9	3	2
UDP-glucose pyrophosphorylase - *Populus tremula* x *Populus tremuloides*	Q5YLM4_9ROSI	51.778/5.68	80	5	2	2
AAA family ATPase, CDC48 subfamily - *Oryza sativa* (japonica cultivar-group)	Q7XE16_ORYSA	90.857/5.07	68	3	2	1
AB004247 NID - *Citrus unshiu* (H+-ATPase catalytic subunit)	BAA87891	68.638/5.29	62	15	10	5
F1-ATPase alpha subunit (Fragment) - *Humbertia madagascariensis*	Q5S817_9ASTE	46.153/5.98	142	20	9	4
H+-transporting two-sector ATPase alpha chain - kidney bean mitochondrion	S26979	55.310/6.51	245	18	9	5
H+-transporting two-sector ATPase beta chain, mitochondrial - Para rubber tree	S20504	60.221/5.95	522	31	24	11
Mitochondrial F1-ATPase gamma subunit - *Ipomoea nil* (Japanese morning glory) (Pharbitis nil)	Q59I53_IPONI	35.370/9.02	75	4	2	1
NADH dehydrogenase subunit F (Fragment) - *Typha angustifolia* (Narrow leaf cattail)	O47212_TYPAN	77.016/9.13	60	2	3	1
Similarity to AAA-type ATPase - *Arabidopsis thaliana* (Mouse-ear cress)	Q9FLD5_ARATH	60.310/6.28	47	2	1	1
Vacuolar ATP synthase catalytic subunit A - *Phaseolus aureus* (Mung bean)	VATA_PHAAU	68.507/5.30	53	1	1	1
DNA and RNA metabolism associated proteins (6)						
Helicase, C-terminal - *Medicago truncatula* (Barrel medic)	Q1SRR4_MEDTR	46.689/5.57	58	14	5	2
Putative DEAD box protein - *Oryza sativa* (japonica cultivar-group)	Q5QLP5_ORYSA	48.291/9.57	55	3	1	1

Putative nuclear ribonuclease Z - *Oryza sativa* (japonica cultivar-group)	Q6H8B1_ORYSA	33.486/6.31	51	5	1	1
Reverse transciptase (Fragment) - *Brassica juncea* (Leaf mustard) (Indian mustard)	Q3MSN9_BRAJU	10.377/9.24	56	10	1	1
Profilin (Allergen Fra a 4) - *Fragaria ananassa* (Strawberry)	PROF_FRAAN	14.058/4.63	54	9	1	1
Maturase-like protein - *Adesmia volckmannii*	Q9TKT4_9FABA	61.146/8.98	53	3	3	1
DNA binding protein (2)						
Histone H2B.10 (HTB2) - *Arabidopsis thaliana* (Mouse-ear cress)	H2B10_ARATH	15.592/10.05	101	18	4	2
Histone H4 - garden pea	HSPM4	11.402/11.48	76	11	2	1
Amino acid metabolism (13)						
5-methyltetrahydropteroyltriglutamate-homocysteine S-methyltransferase - *Madagascar periwinkle*	S57636	84.804/6.10	106	7	7	4
Aspartate aminotransferase P1 - *Lupinus angustifolius* (Narrow-leaved blue lupin)	Q40107_LUPAN	45.827/8.36	105	10	4	2
Aspartate transaminase, cytosolic - rice	JC5124	44.479/7.75	102	6	3	1
Glutamate-ammonia ligase alpha, cytosolic - garden pea	AJPMQA	39.264/6.12	65	4	1	1
Glutamate-ammonia ligase beta - alfalfa	AJAAQ	39.083/5.49	75	4	2	1
Glycine hydroxymethyltransferase - *Arabidopsis thaliana*	B71400	51.685/6.80	68	8	5	2
Glutamine synthetase - *Lactuca sativa*	CAA42689	39.446/5.24	123	8	4	2
Methionine synthase (Fragment) - *Coffea arabica* (Coffee)	Q9M619_COFAR	24.430/5.69	144	13	6	2
Peptidylprolyl isomerase Cyp - kidney bean	S54833	18.148/8.36	170	15	6	1
Peptidylprolyl isomerase ROC1 - *Arabidopsis thaliana*	T06073	18.361/7.68	114	24	2	1
Putative adenosylhomocysteinase - *Trifolium pratense* (Red clover)	Q2PEU5_TRIPR	53.340/5.54	98	18	7	2
Putative alanine aminotransferase (Fragment) - *Arabidopsis thaliana* (Mouse-ear cress)	Q94C83_ARATH	58.415/5.62	61	3	1	1
S-adenosyl-L-methionine synthetase - *Beta vulgaris* (Sugar beet)	Q4H1G4_BETVU	43.189/5.57	106	18	5	2
Protein biosynthesis (27)						
40S ribosomal protein S10-like - *Solanum tuberosum* (Potato)	Q2XPV4_SOLTU	19.830/9.79	73	15	2	1
40S ribosomal protein S3a - *Catharanthus roseus* (Madagascar periwinkle)	RS3A_CATRO	29.503/9.77	56	6	1	1
40S ribosomal protein S3a-like – *Solanum tuberosum* (Potato)	Q2VCH9_SOLTU	29.688/9.76	60	4	1	1
40S ribosomal protein S5 - *Oryza sativa* (japonica cultivar-group)	Q2R4A1_ORYSA	22.193/9.72	65	6	1	1

60S ribosomal protein L12 - *Capsicum annuum* (Bell pepper)	Q6RJY1_CAPAN	17.704/8.81	72	9	1	1
Elongation factor 1, gamma chain - *Medicago truncatula* (Barrel medic)	Q1SL16_MEDTR	47.694/6.43	97	7	4	2
Elongation factor 1-alpha - *Zea mays* (Maize)	O50018_MAIZE	49.259/9.19	127	17	12	5
Elongation factor Tu - *Medicago truncatula* (Barrel medic)	Q1S824_MEDTR	94.123/5.91	80	2	2	1
Eukaryotic initiation factor 4A - *Pennisetum americanum* (Pearl millet)	Q4U474_PENAM	46.992/5.36	51	18	6	2
Gag-pol polyprotein - Populus deltoides (Poplar)	Q710T7_POPDE	153.828/7.85	62	2	2	1
Mitochondrial ribosomal protein L5 - *Medicago truncatula* (Barrel medic)	Q1S6P8_MEDTR	20.672/9.97	50	7	1	1
Peptidase, cysteine peptidase active site; Ribosomal protein L30 - *Medicago truncatula* (Barrel medic)	Q2HVI3_MEDTR	28.486/9.90	68	13	4	1
Proteasome endopeptidase complex iota chain - soybean	T06142	27.375/5.83	51	7	1	1
Proteasome-like protein alpha subunit-like - *Solanum tuberosum* (Potato)	Q2V988_SOLTU	27.068/6.98	80	5	1	1
Putative 40S Ribosomal protein - *Oryza sativa* (Rice)	Q94HF0_ORYSA	33.122/4.86	82	13	4	2
Putative 40S ribosomal protein S6 - *Oryza sativa* (japonica cultivar-group)	Q8LH97_ORYSA	28.455/10.66	66	12	4	2
Putative 40S ribosomal protein S8-like protein - *Solanum tuberosum* (Potato)	Q2XPV9_SOLTU	24.969/10.40	85	19	3	3
Putative 60S ribosomal protein L1 - *Oryza sativa* (japonica cultivar-group)	Q7Y1I5_ORYSA	44.461/10.55	47	3	1	1
Putative eukaryotic translation initiation factor 4A (Fragment) - *Silene latifolia* (Bladder campion)	Q5K407_SILLA	18.654/6.19	52	9	3	1
Ribosomal protein L12 (Fragment) - *Cichorium intybus* (Chicory)	Q9ZSL1_CICIN	15.166/9.88	124	30	3	2
Ribosomal protein L29 - *Medicago truncatula* (Barrel medic)	Q1S449_MEDTR	19.434/10.69	53	7	1	1
Ribosomal protein L6, signature 2 - *Medicago truncatula* (Barrel medic)	Q1T6I0_MEDTR	21.739/9.32	78	7	1	1
Ribosomal protein L7 - *Triticum aestivum* (Wheat)	Q5I7K6_WHEAT	28.103/10.06	51	5	1	1
Ribosomal protein S3 - Norway spruce chloroplast	T11807	25.380/9.62	59	6	2	1
Ribosome inactivating protein 2 (Fragment) - *Zea diploperennis* (Diploperennial teosinte)	Q2XXH0_ZEADI	23.749/6.95	63	5	2	1

Translation elongation factor 1 alpha (Fragment) - *Plantago major* (Common plantain)	Q1EMQ6_PLAMJ	26.997/9.40	51	3	1	1
Translation elongation factor eEF-2 - beet	T14579	93.738/5.93	164	6	9	3
Chaperones (11)						
Chaperonin groEL - castor bean (fragment)	HHCSBA	52.347/4.77	177	15	12	3
Cyclophilin - *Ricinus communis* (Castor bean)	Q8VX73_RICCO	18.142/8.94	191	22	6	3
DnaK protein - *Oryza sativa* (japonica cultivar-group)	Q53RJ5_ORYSA	73.329/5.37	49	2	2	1
DnaK-type molecular chaperone - maize (fragment)	JQ0966	51.657/4.81	99	4	2	1
DnaK-type molecular chaperone precursor, mitochondrial - kidney bean	S25005	72.493/5.95	114	9	5	3
Heat shock protein 70 - *Cucumis sativus* (Cucumber)	Q9M4E6_CUCSA	70.784/5.29	538	29	31	13
Hsp90-2-like - *Solanum tuberosum* (Potato)	Q2XTE5_SOLTU	80.366/5.08	201	7	5	2
Molecular chaperone Hsp90-1 - *Nicotiana benthamiana*	Q6UJX6_NICBE	80.055/4.94	144	16	13	4
Probable chaperonin 60 beta chain - garden pea chloroplast	T06412	62.945/5.85	111	13	8	5
Stromal 70 kDa heat shock-related protein, chloroplast, putative, expressed - *Oryza sativa* (japonica cultivar-group)	Q2QV45_ORYSA	74.041/5.11	57	1	1	1
T-complex protein 1, alpha subunit (Fragment) - *Medicago truncatula* (Barrel medic)	Q1RV32_MEDTR	55.886/6.45	61	3	1	1
Sorting and translocation (2)						
Adenine nucleotide translocator - *Lupinus albus* (White lupin)	O49875_LUPAL	42.134/9.75	94	9	5	3
Pollen coat oleosin-glycine rich protein - *Cardaminopsis arenosa* (Arabidopsis arenosa)	Q6V5C0_CARAS	15.788/10.54	50	7	2	1
Transport (1)						
Putative reversibly glycosylated polypeptide - *Oryza sativa* (japonica cultivar-group)	Q6Z4G3_ORYSA	41.253/6.01	71	7	1	1
Function unknown proteins (10)						
4D11_26 - *Brassica rapa* subsp. pekinensis (Chinese cabbage).	Q4ABW1_BRARP	41.726/11.04	51	4	1	1
AF084478 NID - *Zea mays*	AAC97932	47.908/6.29	65	13	3	1
AF255338 NID - *Glycine max*	AAF70292	25.964/4.70	93	6	1	1
Arabidopsis thaliana genomic DNA, chromosome 5, P1 clone:MEE6 - *Arabidopsis thaliana* (Mouse-ear cress)	Q9FLL1_ARATH	66.648/5.55	81	2	2	1
Hypothetical protein - *Citrus paradisi* (Grapefruit)	O04428_CITPA	32.623/5.46	157	13	5	2

Hypothetical protein OSJNBb0081B07.22 - *Oryza sativa* (japonica cultivar-group)	Q852A3_ORYSA	27.893/6.34	50	6	2	2
Mss4-like - *Medicago truncatula* (Barrel medic)	Q1RSM8_MEDTR	18.126/4.81	87	13	2	1
OSJNBa0067K08.13 protein - *Oryza sativa* (japonica cultivar-group)	Q7XUK3_ORYSA	37.537/6.28	86	5	1	1
Protein At3g24760 - *Arabidopsis thaliana* (Mouse-ear cress)	Q3EB08_ARATH	42.645/5.50	51	5	1	1
Sequence 27 from Patent WO02103001 - Lycopersicon esculentum (Tomato)	CAD70057	42.689/6.11	90	11	3	3
**The total protein number**			**147**			
