# Supplementary material for: Proteome characterization of cassava (Manihot esculenta Crantz) somatic embryos, plantlets and tuberous roots
Source: Proteome Sci. 2010 Feb 27;8:10. doi: 10.1186/1477-5956-8-10 (PMC2842255; doi:10.1186/1477-5956-8-10)
Supplement: Additional file 4 — Table S4. Protein identification in cassava cultivars SC8 tuberous roots. a, MSDB accession number. b, Theoretical molecular mass (kDa) and pI from the MSDB database. c, Probability-based MOWSE (molecular weight search) scores. d, The number of unique peptides identified by MS/MS sequencing, and individual ions scores are all identity or extensive homology (p < 0.05). [file 1477-5956-8-10-S4.PDF]

Additional file 4, Table S4

| Protein name | Accession no[a] | Theoretical molecular mass (KDa)/pI[b] | Score[c] | Sequence coverage (%) | No. of total peptides matched | No. of unique peptides matched[d] |
|---|---|---|---|---|---|---|
| ***Structure (7)*** | | | | | | |
| Actin - *Gossypium hirsutum* (Upland cotton) | Q7XZI7_GOSHI | 41.701/5.31 | 530 | 37 | 20 | 7 |
| Alpha-tubulin 4 (Fragment) - *Gossypium hirsutum* (Upland cotton) | Q8H6L8_GOSHI | 34.013/5.36 | 92 | 13 | 5 | 1 |
| Beta tubulin like protein (Fragment) - *Pyrus pyrifolia* var. culta. | Q3C174_PYRPY | 44.315/5.96 | 144 | 4 | 3 | 2 |
| Fibrillin (Fragment) - *Brassica napus* (Rape) | Q9ZSZ9_BRANA | 25.835/5.12 | 76 | 6 | 1 | 1 |
| Tubulin A - *Glycine max* (Soybean) | Q2TFP2_SOYBN | 49.660/4.99 | 101 | 11 | 4 | 3 |
| tubulin beta chain - *Chlamydomonas reinhardtii* | UBKM | 49.587/4.82 | 57 | 6 | 2 | 1 |
| Putative pectin methylesterase LuPME5 - *Linum usitatissimum* (Flax) (Linseed) | Q94FS5_LINUS | 59.932/8.57 | 57 | 2 | 1 | 1 |
| ***Defense (2)*** | | | | | | |
| Putative DNA-damage-repair/toleration protein DRT102 - *Trifolium pratense* (Red clover) | Q2PEP7_TRIPR | 32.945/5.22 | 70 | 4 | 2 | 1 |
| Thaumatin family - *Oryza sativa* (japonica cultivar-group). | Q7XEP5_ORYSA | 28.347/8.56 | 61 | 8 | 3 | 1 |
| ***Inorganic ion transport and metabolism (2)*** | | | | | | |
| Mitochrondrial voltage-dependent anion-selective channel - *Phaseolus coccineus* (Scarlet runner bean) | Q4PKP6_PHACN | 29.710/8.56 | 54 | 7 | 3 | 1 |
| Outer plastidial membrane protein porin (Voltage-dependent anion- selective channel protein) - *Pisum sativum* (Garden pea) | VDAC_PEA | 29.448/9.11 | 66 | 5 | 2 | 1 |
| ***Detoxifying and antioxidant (11)*** | | | | | | |
| Aldo/keto reductase AKR - *Manihot esculenta* (Cassava) (Manioc) | Q52QX9_MANES | 37.684/6.38 | 90 | 17 | 7 | 3 |
| Ascorbate peroxidase APX3 - *Manihot esculenta* (Cassava) (Manioc) | Q52QX1_MANES | 27.652/5.31 | 286 | 40 | 13 | 4 |
| Catalase CAT1 - *Manihot esculenta* (Cassava) (Manioc) | Q9SW99_MANES | 57.137/6.87 | 50 | 5 | 2 | 1 |
| Ferritin heavy chain precursor - kidney bean | FRFBH | 28.286/5.64 | 76 | 5 | 1 | 1 |
| Glutathione reductase - *Zinnia elegans* (Zinnia) | Q6F4I4_ZINEL | 60.947/8.75 | 69 | 2 | 1 | 1 |

| | | | | | | |
|---|---|---|---|---|---|---|
| Malate oxidoreductase (malic enzyme) [imported] - *Arabidopsis thaliana* | E84582 | 64.238/6.32 | 64 | 6 | 2 | 2 |
| Malic oxidoreductase - *Medicago truncatula* (Barrel medic) | Q1SRX6_MEDTR | 65.296/5.98 | 115 | 3 | 5 | 1 |
| Monodehydroascorbate reductase - *Mesembryanthemum crystallinum* (Common ice plant) | Q93YG1_MESCR | 51.716/6.38 | 75 | 8 | 6 | 2 |
| Monodehydroascorbate reductase I - *Pisum sativum* (Garden pea) | Q66PF9_PEA | 47.321/5.79 | 67 | 7 | 4 | 1 |
| Putative thioredoxin peroxidase (2-Cys peroxiredoxin) - *Oryza sativa* (japonica cultivar-group) | Q6ER94_ORYSA | 28.079/5.67 | 71 | 18 | 6 | 3 |
| Superoxide dismutase (Cu-Zn) - *Arabidopsis thaliana* | DSMUZ | 15.088/5.24 | 73 | 8 | 1 | 1 |
| ***Signal transduction mechanisms (4)*** | | | | | | |
| 14-3-3 f-2 protein (Fragment) - *Nicotiana tabacum* (Common tobacco) | Q75XU9_TOBAC | 27.419/4.80 | 69 | 14 | 4 | 2 |
| 14-3-3 protein - *Manihot esculenta* (Cassava) (Manioc) | Q1AP39_MANES | 29.813/4.75 | 298 | 37 | 12 | 6 |
| Cytokinin binding protein CBP57 - *Nicotiana sylvestris* (Wood tobacco) | Q42939_NICSY | 49.227/6.10 | 67 | 20 | 8 | 1 |
| Putative pollen signalling protein with adenylyl cyclase activity - *Oryza sativa* (japonica cultivar-group) | Q6K3A0_ORYSA | 122.116/5.66 | 50 | 1 | 1 | 1 |
| ***Photosynthesis related proteins (4)*** | | | | | | |
| Putative rubisco subunit binding-protein alpha subunit - *Arabidopsis thaliana* (Mouse-ear cress) | Q8L5U4_ARATH | 62.092/5.05 | 91 | 7 | 5 | 1 |
| Ribulose 1,5-bisphosphate carboxylase/oxygenase (Fragment) - *Pogonatum nipponicum* | Q6YLP8_9BRYO | 40.815/6.33 | 198 | 7 | 5 | 3 |
| Ribulose-1,5-bisphosphate carboxylase/oxygenase large subunit (Fragment) - *Pereskia quisqueyana* | Q3MKL9_9CARY | 50.506/6.11 | 122 | 8 | 7 | 2 |
| RuBisCO subunit binding-protein beta subunit; chaperonin, 60 kDa - *Arabidopsis thaliana* (Mouse-ear cress) | Q9FHA9_ARATH | 63.118/5.73 | 76 | 6 | 5 | 1 |
| ***Carbohydrate and energy metabolism associated proteins (50)*** | | | | | | |
| 1,4-alpha-glucan branching enzyme precursor - *Manihot esculenta* (Cassava) (Manioc) | Q08131_MANES | 96.623/5.38 | 670 | 36 | 50 | 15 |
| 3-phosphoglycerate kinase - *Populus tremuloides* (Quaking aspen) | Q84TL2_POPTM | 42.530/5.98 | 95 | 10 | 3 | 2 |

| | | | | | | |
|---|---|---|---|---|---|---|
| 6-phosphogluconate dehydrogenase (Fragment) - *Zea mays* (Maize) | Q9ZTS5_MAIZE | 9.186/9.00 | 63 | 16 | 2 | 1 |
| Acyl-peptide hydrolase-like - *Arabidopsis thaliana* (Mouse-ear cress) | Q9FG66_ARATH | 75.376/5.08 | 59 | 2 | 1 | 1 |
| ADP-glucose pyrophosphorylase large subunit - *Fragaria ananassa* (Strawberry) | Q6R2I7_FRAAN | 56.411/5.92 | 72 | 2 | 2 | 1 |
| Aldolase (Fragment) - *Triticum aestivum* (Wheat) | Q7X9K7_WHEAT | 23.183/8.90 | 69 | 5 | 1 | 1 |
| Alpha 1,4-glucan phosphorylase L isozyme (Fragment) - *Oryza sativa* (Rice) | Q9ATK9_ORYSA | 104.578/5.38 | 89 | 5 | 7 | 2 |
| beta-glucosidase - cassava | S23940 | 61.334/5.52 | 402 | 25 | 24 | 9 |
| Betaine aldehyde dehydrogenase - *Brassica napus* (Rape) | Q6V4W9_BRANA | 54.701/6.01 | 60 | 5 | 2 | 1 |
| Chloroplast phosphoglycerate kinase - *Populus nigra* (Lombardy poplar) | O82160_POPNI | 50.267/8.54 | 95 | 13 | 5 | 2 |
| Cytoplasmic aldolase - *Oryza sativa* (Rice) | Q40676_ORYSA | 38.695/6.56 | 68 | 8 | 2 | 2 |
| Cytosolic 6-phosphogluconate dehydrogenase - *Oryza sativa* (Rice) | Q7FRX8_ORYSA | 52.688/5.85 | 71 | 11 | 3 | 1 |
| Enolase - *Gossypium barbadense* (Sea-island cotton) (Egyptian cotton) | Q6WB92_GOSBA | 47.702/6.16 | 326 | 34 | 14 | 7 |
| Fructose-bisphosphate aldolase , cytosolic - common ice plant | T12416 | 38.134/6.49 | 195 | 12 | 7 | 3 |
| Glucose-6-phosphate isomerase - *Lycopersicon esculentum* (Tomato) | Q68HC8_LYCES | 67.622/5.49 | 55 | 14 | 5 | 3 |
| Glyceraldehyde-3-phosphate-dehydrogenase - *Lupinus albus* (White lupin) | Q53I52_LUPAL | 32.166/6.80 | 380 | 26 | 13 | 5 |
| Glycogen phosphorylase B; starch phosphorylase - *Arabidopsis thaliana* (Mouse-ear cress) | Q9LIB2_ARATH | 108.517/5.36 | 127 | 4 | 3 | 2 |
| Ketol-acid reductoisomerase (Fragment) - *Platanus acerifolia* (London plane tree) | Q1M2Z5_PLAAC | 18.500/10.47 | 58 | 10 | 1 | 1 |
| Malate dehydrogenase (Fragment)- *Mesembryanthemum crystallinum* (Common ice plant) | Q9ZSQ8_MESCR | 24.174/8.46 | 48 | 6 | 2 | 1 |
| Malate dehydrogenase, cytosolic - common ice plant | T12433 | 35.475/6.00 | 180 | 17 | 10 | 4 |

| | | | | | | |
|---|---|---|---|---|---|---|
| Phosphoglycerate dehydrogenase - *Arabidopsis thaliana* (Mouse-ear cress) | Q9LT69_ARATH | 62.083/8.41 | 63 | 2 | 1 | 1 |
| Phosphoglycerate kinase, putative - *Arabidopsis thaliana* (Mouse-ear cress) | Q8LFV7_ARATH | 42.121/5.49 | 186 | 22 | 8 | 4 |
| Phospholipase D - castor bean | T10171 | 91.934/5.44 | 76 | 9 | 5 | 2 |
| Phospholipase D alpha 2 - *Arachis hypogaea* (Peanut) | Q2HWT8_ARAHY | 91.826/5.54 | 69 | 9 | 6 | 2 |
| Probable malate dehydrogenase - garden pea | T06386 | 41.821/7.62 | 63 | 7 | 4 | 2 |
| Putative 2-oxoglutarate dehydrogenase E2 subunit - *Oryza sativa* (japonica cultivar-group) | Q6K9D8_ORYSA | 49.350/6.77 | 56 | 5 | 2 | 1 |
| Pyruvate kinase - *Medicago truncatula* (Barrel medic) | Q1S8N3_MEDTR | 57.704/6.18 | 157 | 4 | 5 | 1 |
| Pyruvate kinase-like (Fragment) - *Deschampsia antarctica* (Antarctic hairgrass) | Q8LPV6_DESAN | 53.409/6.31 | 69 | 8 | 2 | 1 |
| Sinapyl alcohol dehydrogenase-like protein - *Populus tremula* x Populus tremuloides | Q5I6D6_9ROSI | 38.963/6.23 | 68 | 10 | 2 | 1 |
| Starch branching enzyme I - *Ipomoea batatas* (Sweet potato) (Batate) | Q18PQ2_IPOBA | 99.706/4.94 | 59 | 3 | 2 | 1 |
| Starch phosphorylase isoform L precursor, chloroplast - fava bean | S47243 | 113.220/5.63 | 213 | 12 | 22 | 6 |
| Starch phosphorylase L - potato | S34189 | 110.631/5.20 | 417 | 8 | 20 | 4 |
| Starch phosphorylase precursor - sweet potato | T10947 | 108.452/5.26 | 126 | 6 | 12 | 3 |
| Sucrose synthase (Fragment) - *Manihot esculenta* (Cassava) (Manioc) | Q5PYQ4_MANES | 31.489/5.44 | 276 | 25 | 11 | 4 |
| Transaldolase-like protein - *Solanum tuberosum* (Potato) | Q38HS6_SOLTU | 47.862/5.95 | 220 | 13 | 7 | 3 |
| Transketolase precursor, chloroplast - spinach | T09015 | 80.231/6.20 | 152 | 4 | 6 | 1 |
| Transketolase, chloroplast - *Zea mays* (Maize) | TKTC_MAIZE | 72.948/5.47 | 160 | 5 | 2 | 1 |
| UDP-glucose pyrophosphorylase - *Populus tremula* x *Populus tremuloides* | Q5YLM4_9ROSI | 51.778/5.68 | 100 | 5 | 2 | 2 |
| 26S proteasome regulatory complex ATPase RPT3 - *Zea mays* (Maize) | Q6QP36_MAIZE | 38.805/5.62 | 75 | 5 | 1 | 1 |
| AAA family ATPase, CDC48 subfamily - *Oryza sativa* (japonica cultivar-group) | Q7XE16_ORYSA | 90.857/5.07 | 69 | 5 | 2 | 1 |

| | | | | | | |
|---|---|---|---|---|---|---|
| ATP synthase beta subunit (Fragment) - *Tetracera asiatica* | Q9MTV9_9MAGN | 51.954/5.49 | 52 | 4 | 1 | 1 |
| ATPase F1 alpha subunit (Fragment) - *Ecdeiocolea monostachya* | Q8HFC9_9POAL | 44.470/5.74 | 184 | 5 | 3 | 1 |
| ATP-binding cassette transporter AtABCA1 - *Arabidopsis thaliana* (Mouse-ear cress) | Q8W010_ARATH | 209.091/6.45 | 49 | 0 | 1 | 1 |
| ATP-dependent Clp protease ATP-binding subunit clpA CD4B, chloroplast, putative, expressed - *Oryza sativa* (japonica cultivar-group) | Q2QVG9_ORYSA | 101.954/6.62 | 57 | 2 | 1 | 1 |
| Endopeptidase Clp ATP-binding chain SB100 [similarity] - soybean | T07807 | 101.266/5.85 | 169 | 5 | 5 | 3 |
| H+-transporting two-sector ATPase beta chain, mitochondrial - Para rubber tree | S20504 | 60.221/5.95 | 158 | 24 | 14 | 5 |
| NADH dehydrogenase subunit F (Fragment) - *Typha angustifolia* (Narrow leaf cattail) | O47212_TYPAN | 77.016/9.13 | 61 | 2 | 2 | 1 |
| Putative glucose-6-phosphate isomerase - *Oryza sativa* (japonica cultivar-group) | Q6YXI1_ORYSA | 68.379/5.71 | 72 | 1 | 1 | 1 |
| Putative vacuolar proton-ATPase - *Oryza sativa* (japonica cultivar-group) | Q651T8_ORYSA | 68.426/5.20 | 98 | 8 | 8 | 2 |
| Vacuolar ATPase subunit B - *Mesembryanthemum crystallinum* (Common ice plant) | Q8GUB5_MESCR | 54.092/4.96 | 98 | 5 | 2 | 2 |
| ***DNA and RNA metabolism associated proteins (4)*** | | | | | | |
| Putative retrotransposon protein - *Solanum demissum* (Wild potato) | Q6L3S2_SOLDE | 182.056 | 49 | 0 | 2 | 1 |
| Reverse transcriptase (Fragment) - *Alstroemeria inodora* (Lily). | O49243_ALSIN | 10.427/6.48 | 51 | 18 | 1 | 1 |
| Maturase K - *Metrosideros diffusa* | Q5GIY9_9MYRT | 59.621/9.40 | 46 | 3 | 2 | 1 |
| Maturase-like protein - *Adesmia volckmannii* | Q9TKT4_9FABA | 61.146/8.89 | 57 | 3 | 7 | 1 |
| ***Amino acid metabolism (8)*** | | | | | | |
| Aminotransferases class-I pyridoxal-phosphate-binding site - *Medicago truncatula* (Barrel medic) | Q1RSX0_MEDTR | 49.919/6.95 | 61 | 5 | 2 | 1 |
| Arginase (Fragment) - *Prunus armeniaca* (Apricot). | Q1X8N7_PRUAR | 21.232/5.62 | 58 | 12 | 4 | 1 |
| Aspartate aminotransferase (Fragment)- *Securigera parviflora* | Q93WX7_9FABA | 37.372/8.97 | 69 | 4 | 1 | 1 |

| | | | | | | |
|---|---|---|---|---|---|---|
| Glutamate decarboxylase 2 - *Brassica juncea* (Leaf mustard) (Indian mustard) | Q6Q4I3_BRAJU | 56.053/5.71 | 74 | 11 | 2 | 1 |
| Methionine synthase (Fragment) - *Coffea arabica* (Coffee) | Q9M619_COFAR | 24.430/5.69 | 80 | 13 | 4 | 2 |
| Ornithine carbamoyltransferase OOCT1 - *Canavalia lineata* | Q9SEX4_CANLI | 40.042/8.69 | 56 | 7 | 2 | 1 |
| Putative cysteine protease (Fragment) - *Pisum sativum* (Garden pea) | Q9SC34_PEA | 31.093/7.81 | 60 | 4 | 1 | 1 |
| S-adenosyl-L-methionine synthetase - *Beta vulgaris* (Sugar beet) | Q4H1G4_BETVU | 43.189/5.57 | 215 | 18 | 9 | 4 |
| ***Protein biosynthesis (16)*** | | | | | | |
| 40S ribosomal protein S8 - *Chlamydomonas incerta* | Q1WLZ1_CHLIN | 23.930/10.38 | 81 | 14 | 2 | 2 |
| Chloroplast translational elongation factor Tu - *Pelargonium graveolens* (rose geranium) | Q9AXU2_9ROSI | 51.283/6.12 | 56 | 7 | 2 | 1 |
| Elongation factor (Fragment) - *Triticum aestivum* (Wheat) | Q7XYB6_WHEAT | 184.67/5.82 | 93 | 9 | 2 | 1 |
| Elongation factor 1, gamma chain - *Medicago truncatula* (Barrel medic) | Q1SL16_MEDTR | 47.694/6.43 | 92 | 4 | 2 | 1 |
| Elongation factor 1-alpha - *Zea mays* (Maize) | O50018_MAIZE | 49.259/9.19 | 95 | 9 | 6 | 2 |
| Elongation factor Tu - *Medicago truncatula* (Barrel medic) | Q1S824_MEDTR | 94.123/5.91 | 108 | 2 | 5 | 1 |
| Putative elongation factor 1 beta - *Hordeum vulgare* (Barley) | Q9M3U8_HORVU | 24.545/4.52 | 59 | 5 | 2 | 1 |
| Putative elongation factor 2 - *Oryza sativa* (japonica cultivar-group) | Q6H4L2_ORYSA | 93.961/5.85 | 73 | 6 | 3 | 1 |
| Putative nicotiana eukaryotic translation initiation factor 4A - *Oryza sativa* (japonica cultivar-group) | Q5VNM3_ORYSA | 45.570/5.98 | 49 | 3 | 1 | 1 |
| Ribosomal protein S15 - rye chloroplast | A34435 | 10.814/11.23 | 48 | 11 | 1 | 1 |
| Ribosomal protein S3 - Norway spruce chloroplast | T11807 | 25.380/9.62 | 61 | 6 | 1 | 1 |
| Ribosomal protein S5, bacterial and organelle form - *Medicago truncatula* (Barrel medic) | Q1SLE0_MEDTR | 30.431/10.32 | 51 | 4 | 2 | 1 |
| Translation elongation factor eEF-2 - beet | T14579 | 93.738/5.93 | 180 | 5 | 5 | 1 |
| Translation elongation factor EF-Tu precursor, chloroplast - garden pea | T06821 | 53.017/6.62 | 67 | 3 | 2 | 1 |
| Translation factor - *Medicago truncatula* (Barrel medic) | Q1S825_MEDTR | 94.081/5.80 | 362 | 5 | 8 | 1 |
| Translation initiation factor - *Zea mays* (Maize) | O24558_MAIZE | 46.976/5.38 | 69 | 5 | 2 | 2 |

| | | | | | | |
|---|---|---|---|---|---|---|
| ***Chaperones (10)*** | | | | | | |
| Cell-autonomous heat shock cognate protein 70 - *Cucurbita maxima* (Pumpkin) (Winter squash) | Q8GSN2_CUCMA | 71.211/5.17 | 104 | 12 | 5 | 1 |
| Chaperonin groEL - castor bean (fragment) | HHCSBA | 52.347/4.77 | 251 | 4 | 6 | 1 |
| Cytosolic class I small heat shock protein 1B (Fragment) - *Nicotiana tabacum* (Common tobacco) | Q53E42_TOBAC | 15.631/5.39 | 118 | 24 | 14 | 2 |
| DnaK-type molecular chaperone hsc70-3 - tomato | JC4786 | 71.470/5.18 | 277 | 16 | 10 | 5 |
| Heat shock protein - *Pisum sativum* (Garden pea) | Q8H1A6_PEA | 18.044/5.82 | 97 | 18 | 4 | 2 |
| Heat shock protein 17.0 - white spruce | T09253 | 17.057/5.76 | 64 | 10 | 1 | 1 |
| Heat shock protein, 70K, chloroplast - cucumber | T10248 | 75.366/5.15 | 365 | 15 | 11 | 6 |
| Heat-shock protein 80 (Fragment) - *Euphorbia esula* (Leafy spurge) | Q9M5X2_EUPES | 36.229/5.00 | 96 | 6 | 3 | 1 |
| High molecular weight heat shock protein - *Malus domestica* (Apple) (Malus sylvestris) | Q9M6R1_MALDO | 71.171/5.17 | 515 | 35 | 35 | 12 |
| Putative chaperonin 60 beta - *Oryza sativa* (japonica cultivar-group) | Q9LWT6_ORYSA | 64.046/5.60 | 137 | 8 | 6 | 2 |
| ***Sorting and translocation (2)*** | | | | | | |
| Pollen coat oleosin-glycine rich protein - *Cardaminopsis arenosa* (Arabidopsis arenosa) | Q6V5C0_CARAS | 157.88/10.54 | 48 | 7 | 1 | 1 |
| Putative adenine nucleotide translocase (Fragment) - *Castanea sativa* (Sweet chestnut) | Q945C9_CASSA | 13.023/9.70 | 145 | 20 | 2 | 2 |
| ***Transport (3)*** | | | | | | |
| AGTHI4 NID - *Alnus glutinosa* (thiazole biosynthetic enzyme) | CAA66064 | 37.041/5.42 | 160 | 11 | 7 | 1 |
| Putative TPR repeat nuclear phosphoprotein - *Arabidopsis thaliana* (Mouse-ear cress) | Q8VYL2_ARATH | 83.262/5.45 | 47 | 2 | 2 | 1 |
| Thiamin biosynthesis protein thi4 - *Arabidopsis thaliana* | S71191 | 36.641/5.82 | 272 | 20 | 11 | 4 |
| ***Function unknown proteins (32)*** | | | | | | |
| 52O08_40 - *Brassica rapa* subsp. pekinensis (Chinese cabbage) | Q4ABR7_BRARP | 31.073/5.50 | 61 | 6 | 1 | 1 |
| AB013353 NID - *Pyrus pyrifolia* | BAA25917 | 51.813/5.99 | 55 | 3 | 1 | 1 |

| | | | | | | |
|---|---|---|---|---|---|---|
| AC007190 NID - *Arabidopsis thaliana* | AAF19533 | 69.149/6.01 | 47 | 2 | 5 | 1 |
| AF255338 NID - *Glycine max* | AAF70292 | 25.964/4.70 | 93 | 6 | 2 | 1 |
| Arabidopsis thaliana genomic DNA, chromosome 5, P1 clone:MEE6 - *Arabidopsis thaliana* (Mouse-ear cress) | Q9FLL1_ARATH | 66.684/5.55 | 60 | 2 | 7 | 1 |
| At5g19300 - *Arabidopsis thaliana* (Mouse-ear cress) | Q6NLH7_ARATH | 44.852/6.09 | 51 | 2 | 2 | 1 |
| DP000009 NID - *Oryza sativa* (japonica cultivar-group) | ABF95258 | 71.056/5.10 | 78 | 6 | 2 | 1 |
| F21J9.11 (At1g24450/F21J9_210) - *Arabidopsis thaliana* (Mouse-ear cress) | Q9FYL8_ARATH | 20.729/9.51 | 89 | 9 | 2 | 1 |
| Hypothetical protein - *Citrus paradisi* (Grapefruit) | O04428_CITPA | 32.623/5.46 | 137 | 13 | 5 | 2 |
| Hypothetical protein - *Medicago truncatula* (Barrel medic) | Q1SCA0_MEDTR | 50.722/8.08 | 59 | 2 | 1 | 1 |
| hypothetical protein - wild cabbage (fragment) | T14439 | 28.896/4.94 | 49 | 14 | 3 | 1 |
| Hypothetical protein (Fragment) - *Cicer arietinum* (Chickpea) (Garbanzo) | Q9LEN5_CICAR | 54.901/4.87 | 72 | 10 | 4 | 2 |
| hypothetical protein At2g11600 [imported] - *Arabidopsis thaliana* | F84498 | 45.208/5.74 | 51 | 4 | 1 | 1 |
| hypothetical protein At2g36420 [imported] - *Arabidopsis thaliana* | E84780 | 50.560/4.95 | 47 | 2 | 2 | 1 |
| Hypothetical protein At3g62360 - *Arabidopsis thaliana* (Mouse-ear cress) | Q56XW4_ARATH | 60.296/6.82 | 51 | 2 | 1 | 1 |
| Hypothetical protein B1249E06.27 - *Oryza sativa* (japonica cultivar-group) | Q5QL76_ORYSA | 15.588/5.28 | 50 | 11 | 1 | 1 |
| hypothetical protein F13K23.2 - *Arabidopsis thaliana* | A86261 | 72.277/6.15 | 49 | 3 | 4 | 1 |
| Hypothetical protein F14O10.13 (Vacuolar-type H+-ATPase subunit B3) (VHA-B3) (Hypothetical protein At1g20260) - *Arabidopsis thaliana* (Mouse-ear cress) | Q8W4E2_ARATH | 54.278/4.99 | 48 | 4 | 2 | 1 |
| hypothetical protein F25G13.100 - *Arabidopsis thaliana* | T10203 | 34.414/9.05 | 66 | 6 | 4 | 1 |
| Hypothetical protein P0685E10.8 - *Oryza sativa* (japonica cultivar-group) | Q65XS1_ORYSA | 75.324/6.49 | 58 | 2 | 2 | 1 |
| Hypothetical protein T9J14.9 - *Arabidopsis thaliana* (Mouse-ear cress) | Q9CAW0_ARATH | 70.226/5.20 | 49 | 1 | 2 | 1 |
| OSJNBa0067K08.13 protein - *Oryza sativa* (japonica cultivar-group) | Q7XUK3_ORYSA | 37.537/6.28 | 111 | 13 | 3 | 1 |

| | | | | | | |
|---|---|---|---|---|---|---|
| OSJNBa0070C17.13 protein - *Oryza sativa* (japonica cultivar-group) | Q7F9B4_ORYSA | 43.068/8.34 | 112 | 8 | 3 | 1 |
| OSJNBb0059K02.15 protein - *Oryza sativa* (japonica cultivar-group) | Q7XMP6_ORYSA | 63.925/6.83 | 171 | 3 | 4 | 1 |
| OSJNBb0116K07.9 protein - *Oryza sativa* (japonica cultivar-group) | Q7F8Y3_ORYSA | 53.412/7.01 | 57 | 2 | 2 | 1 |
| Protein At1g79930 - *Arabidopsis thaliana* (Mouse-ear cress) | Q2V4B7_ARATH | 87.262/5.19 | 49 | 1 | 2 | 1 |
| protein F1N21.10 [imported] - *Arabidopsis thaliana* | E96696 | 39.998/6.97 | 78 | 8 | 2 | 1 |
| Putative RUSH-1alpha - *Oryza sativa* (japonica cultivar-group) | Q8GSA1_ORYSA | 91.274/8.98 | 52 | 1 | 2 | 1 |
| Putative tetratricoredoxin - *Oryza sativa* (japonica cultivar-group) | Q6ES52_ORYSA | 34.959/5.84 | 57 | 3 | 1 | 1 |
| PWI245645 NID - *Prototheca wickerhamii* | CAB53113 | 44.679/6.54 | 53 | 8 | 2 | 1 |
| Sequence 5 from Patent WO0032789 - *Fragaria ananassa* (Strawberry) | CAC09050 | 43.622/8.03 | 61 | 6 | 3 | 1 |
| U-box domain containing protein, expressed - *Oryza sativa* (japonica cultivar-group) | Q2QU09_ORYSA | 92.989/6.02 | 49 | 2 | 1 | 1 |
| **The total protein number** | | | **155** | | | |
