# Supplementary material for: Proteome characterization of cassava (Manihot esculenta Crantz) somatic embryos, plantlets and tuberous roots
Source: Proteome Sci. 2010 Feb 27;8:10. doi: 10.1186/1477-5956-8-10 (PMC2842255; doi:10.1186/1477-5956-8-10)
Supplement: Additional file 5 — Figure S1. Results of mitochrondrial voltage-dependent anion-selective channel in the adventitious roots of cassava cultivar SC8 as a representation using LC-ESI-MS/MS. A, protein view and B, peptide view of Mascot search results. [file 1477-5956-8-10-S5.PDF]

Additional file 5, Figure S1

Mascot Search Results

A

Protein View

Match to: **Q4PKP6_PHACN** Score: **77**
Mitochrondrial voltage-dependent anion-selective channel.- Phaseolus coccineus (Scarlet runner bean).
Found in search of H:\Songbi-Protein identification by LC MS MS 2009\Songbi LC MS MS 2009 03 20 (HeLa lamin A-C IP and cassava)\Songbi Sample R8_LCA_1B,8_01_11052.d\Analysis.mgf

Nominal mass (M_r): **29710**; Calculated pI value: **8.56**

NCBI BLAST search of Q4PKP6_PHACN against nr
Unformatted sequence string for pasting into other applications

Taxonomy: Phaseolus coccineus
Links to retrieve other entries containing this sequence from NCBI Entrez:
AAY82249 from Phaseolus coccineus

Variable modifications: Carbamidomethyl (C),Oxidation (M)
Cleavage by Trypsin: cuts C-term side of KR unless next residue is P
Sequence Coverage: **7%**

Matched peptides shown in **Bold Red**

```
  1 MAKGPGLYTD IGKKARDLLF KDYHSDQKFT VTTYSPTGVA ITSSGTRKGE
 51 LFLADVNTQL KNKNITTDIK VDTDSNLFTT ITVNEPAPGL KAIFNFKVPD
101 QRSGKVELQY LHDYAGISTS VGLTANPIVN FSGVVGTNIL ALGADLSFDT
151 KIGELTKSNA GLSFTKDDLI ASLTLNDKGD ALNAAYYHVV NPLTNTAVGA
201 EVTHRFSTNE NTITLGTQHA LDPLTTLKAR VNNFGKSSAL IQHEWRPKSF
251 FTISGEVDTK AIEKSAKVGL SLVLKP
```

Show predicted peptides also

Sort Peptides By ○ Residue Number ○ Increasing Mass ○ Decreasing Mass

Start - End	Observed	Mr(expt)	Mr(calc)	Delta	Miss	Sequence
71 - 91	1116.1240	2230.2334	2231.1322	-0.8987	0	K.VDTDSNLFTTITVNEPAPGLK.A (Ions score

77)

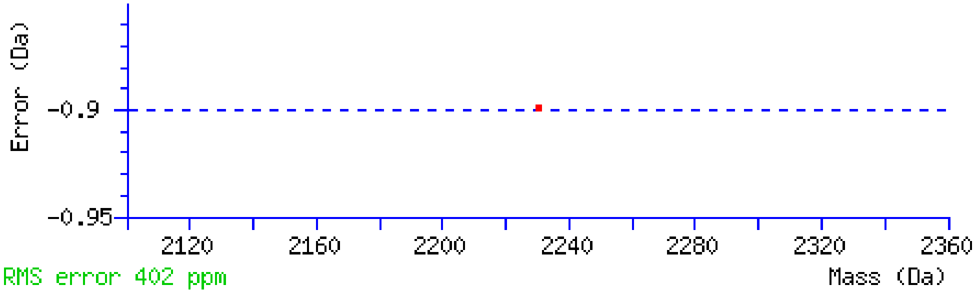

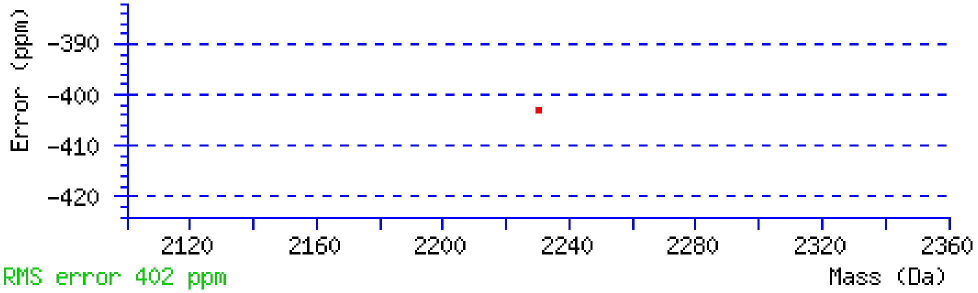

>P1;Q4PKP6_PHACN Mitochrondrial voltage-dependent anion-selective channel.- Phaseolus coccineus (Scarlet runner bean). C;Species Q4PKP6_PHACN: Phaseolus coccineus (Scarlet runner bean). C;Species AAY82249: Phaseolus coccineus C;Family: Eukaryota; Viridiplantae; Streptophyta; Embryophyta; Tracheophyta; Spermatophyta; Magnoliophyta; eudicotyledons; core eudicotyledons; rosids; eurosids I; Fabales; Fabaceae; Papilionoideae; Phaseoleae; Phaseolus. C;Accession: Q4PKP6; C;Reference [1] C;NUCLEOTIDE SEQUENCE. R; Smeyers M., El Jaziri M., Homble F.; Submitted (MAY-2005) to the EMBL/GenBank/DDBJ databases. C;19-JUL-2005, integrated into UniProtKB/TrEMBL. C;19-JUL-2005, sequence version 1. C;07-FEB-2006, entry version 3. C;--- C;Copyrighted by the UniProt Consortium, see http://www.uniprot.org/terms C;Distributed under the Creative Commons Attribution-NoDerivs License C;--- C;EMBL; DQ072165; AAY82249.1; -; mRNA. C;GO; GO:0005741; C:mitochondrial outer membrane; IEA. C;GO; GO:0008308; F:voltage-gated ion-selective channel activity; IEA. C;GO; GO:0006820; P:anion transport; IEA. C;InterPro; IPR001925; Porin_Euk. C;Pfam; PF01459; Porin_3; 1. C;PROSITE; PS00558; EUKARYOTIC_PORIN; 1. C;Keywords: C; SRCDB TREMBL C;IDN_GENBANK AAY82249;

Mascot: http://www.matrixscience.com/

B

Mascot Search Results

Peptide View

MS/MS Fragmentation of **VDTDSNLFTTITVNEPAPGLK**
Found in **Q4PKP6_PHACN**, Mitochrondrial voltage-dependent anion-selective channel.- Phaseolus coccineus (Scarlet runner bean).

Match to Query 282: 2230.233448 from(1116.124000,2+) intensity(9467.0000)
Title: Cmpd 274, +MSn(1116.11) 30.5 min
Data file H:\Songbi-Protein identification by LC MS MS 2009\Songbi LC MS MS 2009 03 20 (HeLa lamin A-C IP and cassava)\Songbi Sample R8_LCA_1B,8_01_11052.d\Analysis.mgf

Click mouse within plot area to zoom in by factor of two about that point
Or, Plot from 400 to 1500 Da Full range

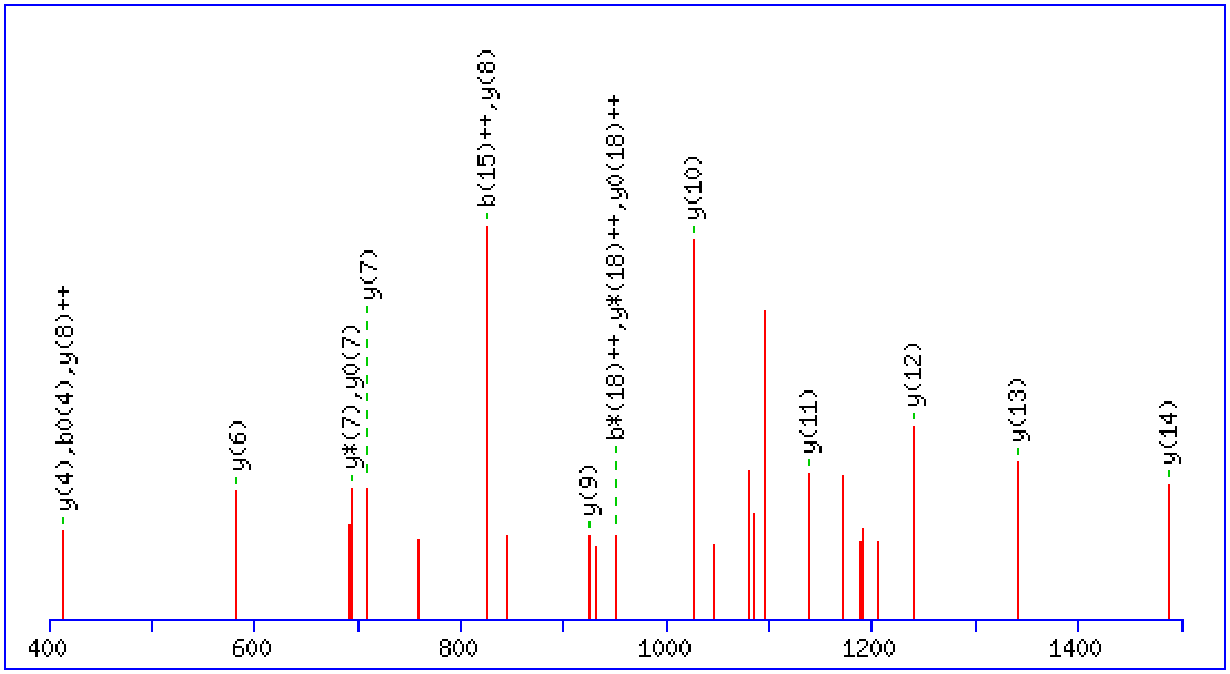

Monoisotopic mass of neutral peptide Mr(calc): 2231.1322
Ions Score: 77 **Expect:** 4.1e-05
Matches (Bold Red): 18/216 fragment ions using 19 most intense peaks

#	b	b^{++}	b^{*}	b^{*++}	b^{0}	b^{0++}	Seq.	y	y^{++}	y^{*}	y^{*++}	y^{0}	y^{0++}	#
1	100.0757	50.5415					V							21
2	215.1026	108.0550			197.0921	99.0497	D	2133.0710	1067.0391	2116.0445	1058.5259	2115.0604	1058.0339	20
3	316.1503	158.5788			298.1397	149.5735	T	2018.0441	1009.5257	2001.0175	1001.0124	2000.0335	1000.5204	19
4	431.1773	216.0923			**413.1667**	207.0870	D	1916.9964	959.0018	1899.9698	**950.4886**	1898.9858	**949.9965**	18
5	518.2093	259.6083			500.1987	250.6030	S	1801.9694	901.4884	1784.9429	892.9751	1783.9589	892.4831	17
6	632.2522	316.6297	615.2257	308.1165	614.2416	307.6245	N	1714.9374	857.9723	1697.9109	849.4591	1696.9268	848.9671	16
7	745.3363	373.1718	728.3097	364.6585	727.3257	364.1665	L	1600.8945	800.9509	1583.8679	792.4376	1582.8839	791.9456	15
8	892.4047	446.7060	875.3781	438.1927	874.3941	437.7007	F	**1487.8104**	744.4088	1470.7839	735.8956	1469.7999	735.4036	14
9	993.4524	497.2298	976.4258	488.7165	975.4418	488.2245	T	**1340.7420**	670.8746	1323.7155	662.3614	1322.7314	661.8694	13
10	1094.5000	547.7537	1077.4735	539.2404	1076.4895	538.7484	T	**1239.6943**	620.3508	1222.6678	611.8375	1221.6838	611.3455	12
11	1207.5841	604.2957	1190.5576	595.7824	1189.5735	595.2904	I	**1138.6467**	569.8270	1121.6201	561.3137	1120.6361	560.8217	11
12	1308.6318	654.8195	1291.6052	646.3063	1290.6212	645.8142	T	**1025.5626**	513.2849	1008.5360	504.7717	1007.5520	504.2796	10
13	1407.7002	704.3537	1390.6737	695.8405	1389.6896	695.3485	V	**924.5149**	462.7611	907.4884	454.2478	906.5043	453.7558	9
14	1521.7431	761.3752	1504.7166	752.8619	1503.7326	752.3699	N	**825.4465**	**413.2269**	808.4199	404.7136	807.4359	404.2216	8
15	1650.7857	**825.8965**	1633.7592	817.3832	1632.7752	816.8912	E	**711.4036**	356.2054	**694.3770**	347.6921	**693.3930**	347.2001	7
16	1747.8385	874.4229	1730.8119	865.9096	1729.8279	865.4176	P	**582.3610**	291.6841	565.3344	283.1709			6
17	1818.8756	909.9414	1801.8491	901.4282	1800.8650	900.9362	A	485.3082	243.1577	468.2817	234.6445			5
18	1915.9284	958.4678	1898.9018	**949.9545**	1897.9178	949.4625	P	**414.2711**	207.6392	397.2445	199.1259			4
19	1972.9498	986.9786	1955.9233	978.4653	1954.9393	977.9733	G	317.2183	159.1128	300.1918	150.5995			3
20	2086.0339	1043.5206	2069.0073	1035.0073	2068.0233	1034.5153	L	260.1969	130.6021	243.1703	122.0888			2
21							K	147.1128	74.0600	130.0863	65.5468			1

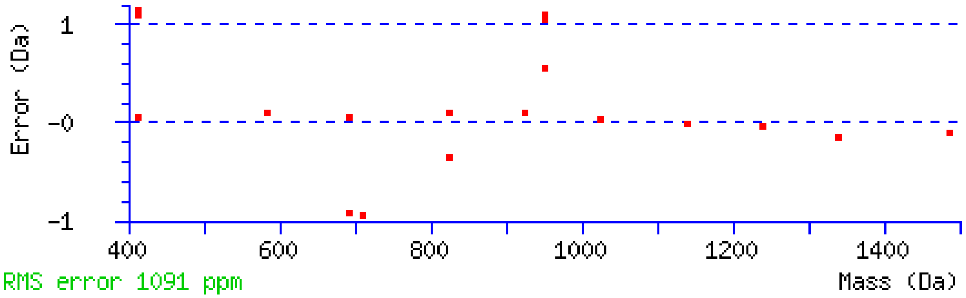

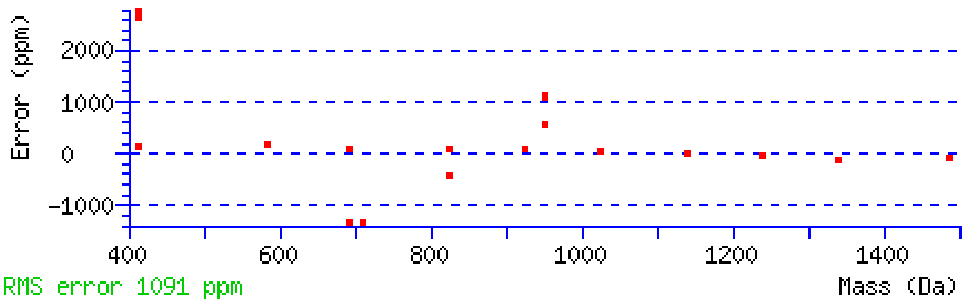

NCBI **BLAST** search of VDTDSNLFTTITVNEPAPGLK
(Parameters: blastp, nr protein database, expect=20000, no filter, PAM30)
Other BLAST web gateways

All matches to this query

Score	Mr(calc):	Delta	Sequence
76.5	2231.1322	-0.8987	VDTDSNLFTTITVNEPAPGLK
76.5	2231.1322	-0.8987	VDTNSNLFTTITVDEPAPGLK
66.4	2233.1114	-2.8780	VDTNSNVYTTITVDEPAPGLK
32.1	2231.1256	-0.8921	LPRLSDMYTLTIASADPHSK
21.6	2229.9802	0.2533	FETDGFEVVDTPLTTDSDDK
19.5	2230.0980	0.1354	TFVFSWGEATITLEDVMLR
19.3	2228.1181	2.1154	LDTPNATMNPDAVLVQSLMAK
19.2	2232.0682	-1.8348	CHDAPILMAIPLIFLALXXS
18.7	2228.1033	2.1301	QPERVVDSDGGSGGSVTVEVVR
18.0	2228.9621	1.2713	GTQPRAMLGIPCYCCVEGCR

Mascot: http://www.matrixscience.com/
