# Supplementary figures and images for: Proteome characterization of cassava (Manihot esculenta Crantz) somatic embryos, plantlets and tuberous roots
Source: Proteome Sci. 2010 Feb 27;8:10. doi: 10.1186/1477-5956-8-10 (PMC2842255; doi:10.1186/1477-5956-8-10)

## Additional file 7, Figure S2

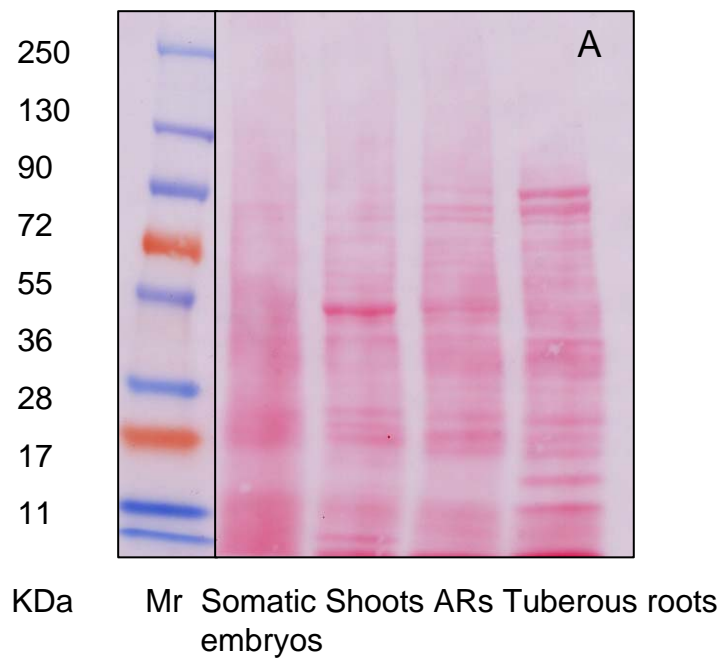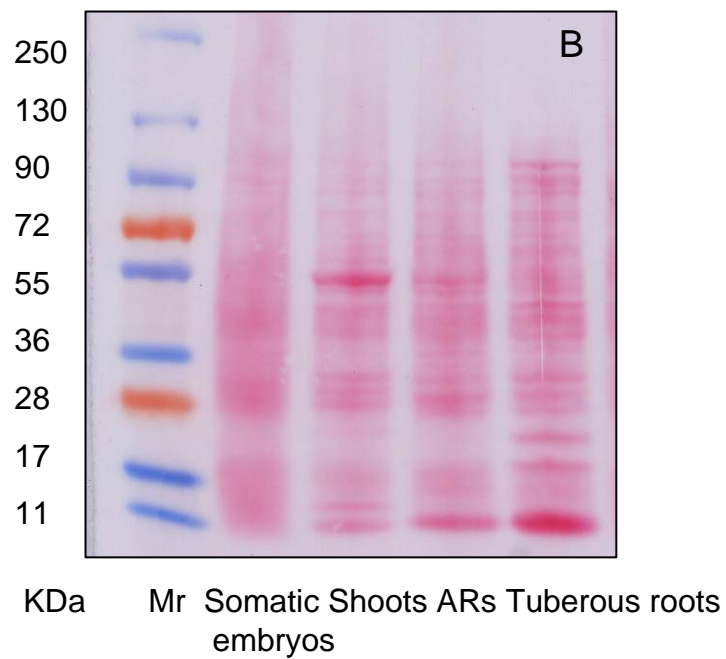

Supplement: Additional file 7 — Figure S2. Proteins on nitrocellulose membranes were detected with Ponceau staining. Proteins transferred from SDS-PAGE gels to nitrocellulose membranes were stained with 5% Ponceau in 1% acetic acid for several minutes, and then washed with 1% acetic acid. A, For Rubisco detection with antiRubisco-polyclonal antibody and B, For α-tubulan detection with anti-α-tubulin-monoclonal antibody. ARs, adventitious roots. [file 1477-5956-8-10-S7.PDF]

Additional file 8, Figure S3

A

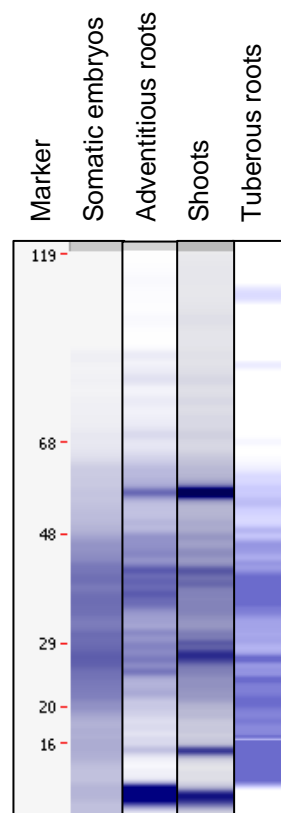

B

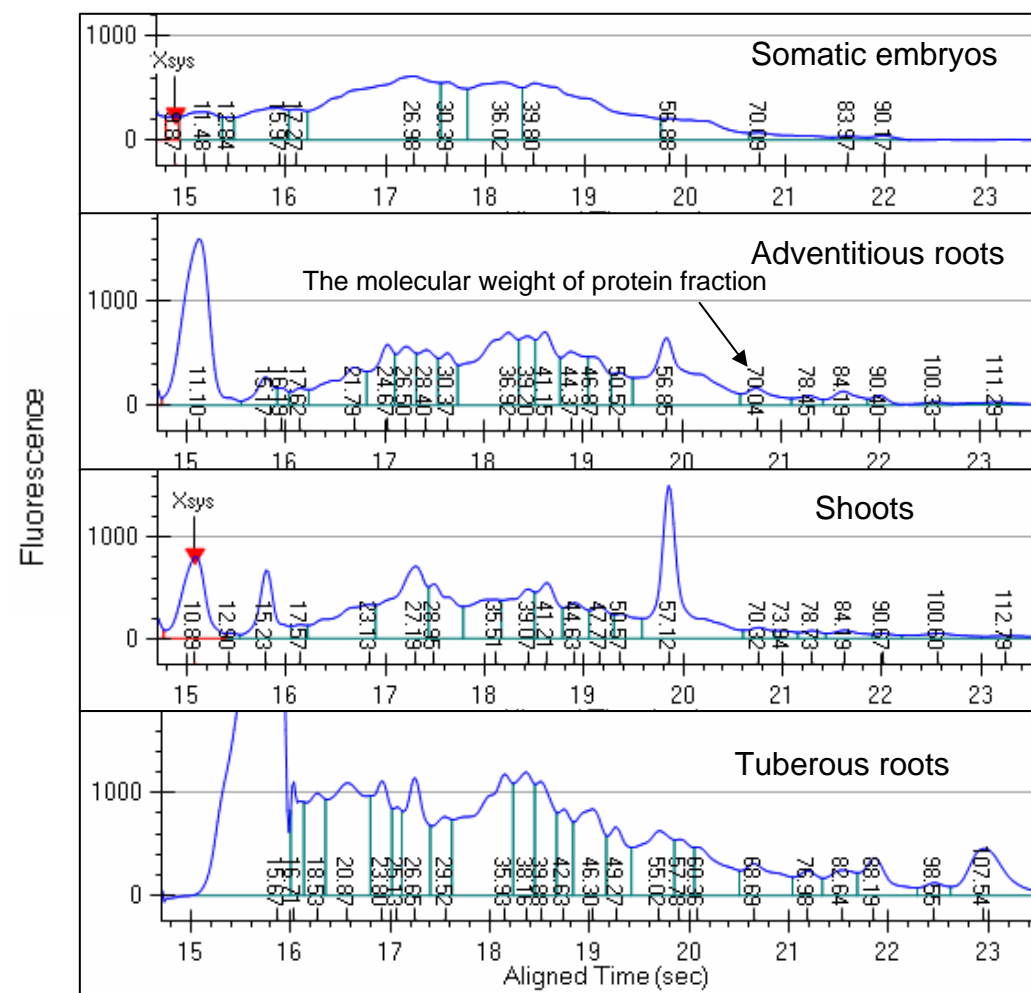

Supplement: Additional file 8 — Figure S3. Analysis of protein fractions by LabChip GXII Electrophoresis System. A, Protein fraction patterns of cassava cultivar SC8 somatic embryos, plantlets (adventitious roots and shoots) and tuberous roots. The left lane represents molecular mass markers. B, Protein fraction intensity detected by fluorescence analysed with LabChipGX program (Version 1.1.119.0). The arrow presents the molecular weight of one protein fraction. [file 1477-5956-8-10-S8.PDF]
